# Designing image segmentation studies: Statistical power, sample size and reference standard quality

**DOI:** 10.1016/j.media.2017.07.004

**Published:** 2017-12

**Authors:** Eli Gibson, Yipeng Hu, Henkjan J. Huisman, Dean C. Barratt

**Affiliations:** a Department of Radiology, Radboud University Medical Center, Nijmegen, The Netherlands; b Department of Medical Physics and Biomedical Engineering, University College London, London, United Kingdom; c Centre for Medical Image Computing, The Engineering Front Building, University College London, Malet Place, London, WC1E 6BT, United Kingdom

**Keywords:** Image segmentation, Segmentation accuracy, Statistical power, Reference standard

## Abstract

Segmentation algorithms are typically evaluated by comparison to an accepted reference standard. The cost of generating accurate reference standards for medical image segmentation can be substantial. Since the study cost and the likelihood of detecting a clinically meaningful difference in accuracy both depend on the size and on the quality of the study reference standard, balancing these trade-offs supports the efficient use of research resources.

In this work, we derive a statistical power calculation that enables researchers to estimate the appropriate sample size to detect clinically meaningful differences in segmentation accuracy (i.e. the proportion of voxels matching the reference standard) between two algorithms. Furthermore, we derive a formula to relate reference standard errors to their effect on the sample sizes of studies using lower-quality (but potentially more affordable and practically available) reference standards.

The accuracy of the derived sample size formula was estimated through Monte Carlo simulation, demonstrating, with 95% confidence, a predicted statistical power within 4% of simulated values across a range of model parameters. This corresponds to sample size errors of less than 4 subjects and errors in the detectable accuracy difference less than 0.6%. The applicability of the formula to real-world data was assessed using bootstrap resampling simulations for pairs of algorithms from the PROMISE12 prostate MR segmentation challenge data set. The model predicted the simulated power for the majority of algorithm pairs within 4% for simulated experiments using a high-quality reference standard and within 6% for simulated experiments using a low-quality reference standard. A case study, also based on the PROMISE12 data, illustrates using the formulae to evaluate whether to use a lower-quality reference standard in a prostate segmentation study.

## Highlights

• A sample size calculation for segmentation accuracy studies is derived.
• Parameters include accuracy difference, algorithm disagreement and a design factor.
• A formula is derived to account for errors in the study reference standard.
• A case study illustrates the application of the theory to a segmentation study design.

## Graphical abstract

**Figure:**
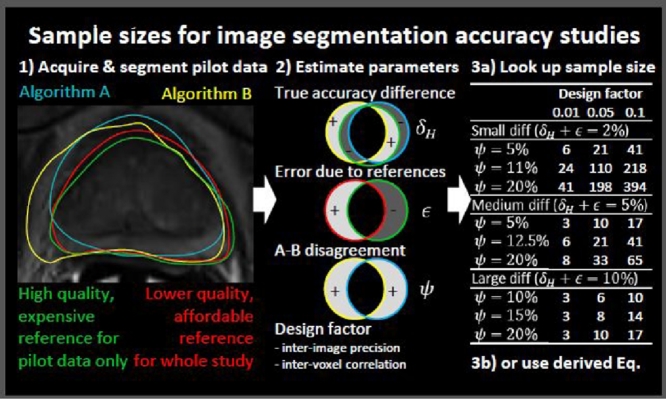

## 1 Introduction

Demonstrating an improvement in segmentation algorithm accuracy typically involves comparison with an accepted reference standard, such as manual expert segmentations or other imaging modalities (e.g. histology). In many medical image segmentation problems, such segmentations are challenging due to the variable appearance of anatomical/pathological features, ambiguous anatomical definitions, clinical constraints, and interobserver variability. The resulting errors in the reference standards introduce errors in the performance measures used to compare segmentation algorithms, and can impact the probability of detecting a significant difference between algorithms, referred to as the statistical power (Beiden et al., 2000).

**Fig. 1:**
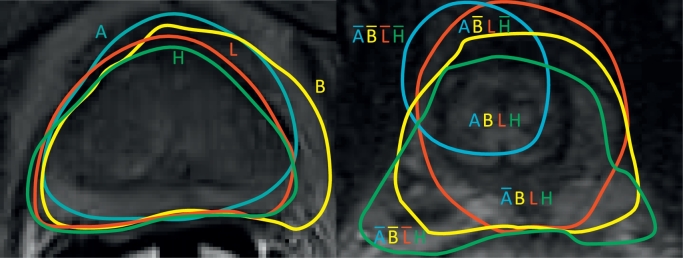
Left: Illustrative prostate MRI segmentations from the PROMISE12 prostate segmentation challenge (Litjens et al., 2014b) by two algorithms – A (blue) and B (yellow) – and the two manually contoured reference standards – L (red) which is of lower quality and H (green) that is of higher quality. Compared to H, L oversegmented anteriorly where image information was ambiguous, affecting accuracy measurements of A and B using L. Right: Harder apical segmentations showing regions containing voxels with different combinations of segmentation labels ABLH (overbar denotes negative classifications). The statistical model underlying the derived sample size formula for segmentation evaluation studies is derived from probability distributions of these voxel-wise segmentation labels. (For interpretation of the references to colour in this figure legend, the reader is referred to the web version of this article.)

The cost and quality of a reference standard is affected by the time and effort devoted to segmentation accuracy, the sample size, and the number, background, experience and proficiency of the observers. For example, the PROMISE12 prostate MRI segmentation challenge used two reference standards (illustrated in Fig. 1): a *high-quality* reference standard manually segmented by one experienced clinical reader and verified by another independent clinical reader, and a *low-quality* reference standard segmented by a less experienced non-clinical observer. An alternative approach is to estimate a high-quality reference standard by combining independent segmentations from multiple observers using algorithms such as STAPLE (Warfield et al., 2004) and SIMPLE (Langerak et al., 2010). A third approach is to mitigate the errors in a lower-quality reference standard by increasing the sample size (Konyushkova, Sznitman, Fua, 2015, Top, Hamarneh, Abugharbieh, 2011, Maier-Hein, Mersmann, Kondermann, Bodenstedt, Sanchez, Stock, Kenngott, Eisenmann, Speidel, 2014, Irshad, Montaser-Kouhsari, Waltz, Bucur, Nowak, Dong, Knoblauch, Beck, 2015). All three of these approaches, however, raise the cost of generating the reference standard, both logistically and economically.

There are clear trade-offs between the sample size of the study, the cost of generating the reference standard, and the reference standard quality. The optimal balance of these trade-offs depends on the relationship between the study design parameters and statistical power. However, standard power calculation formulae do not, in general, account for the quality of reference standard segmentations. Thus, there is a need for new formulae to quantify these relationships. As a first step towards this goal, this paper presents a new sample size calculation relating statistical power to the quality of a reference standard (measured with respect to a higher-quality reference standard). Such a formula can answer key questions in study design:

• **How many validation images are needed to evaluate a segmentation algorithm?**
• **How accurate does the reference standard need to be?**

In preliminary work (Gibson et al., 2015), we derived a relationship between statistical power and the quality of a reference standard for a simplified model that cannot account for correlation between voxels, and made a strong assumption that the reference and algorithm segmentation labels are conditionally independent given the high-quality reference standard. In the present paper, we build on our initial work to develop a generalized model that takes into account the correlation between voxels and the statistical dependence between algorithms and reference standards observed in segmentation studies.

The remainder of this paper outlines the derivation (Section 2.3), application (Sections 3 and 6) and validation (Sections 4 and 5) of a statistical power formula for image segmentation. Insights and heuristics derived from the formula and its validation, as well as limitations of the work, are discussed in Section 7. Appendix A and Appendix B present mathematical details of the derivations.

## 2 Sample size calculations in segmentation evaluation studies

The probability of a study correctly detecting a true effect depends in part on the sample size. A study with a sample size that is too small has a higher risk of missing a meaningful underlying difference, while one with a sample size that is too large may be more expensive than necessary. Sample size calculations relate the probability of a study correctly detecting a true effect to specified and estimated parameters of the study design (Mace, 1964). The sample size depends on the probability distribution of the test statistic under the null and alternate hypotheses. This distribution, in turn, depends on the statistical analysis being performed and on an assumed statistical model of the studied population.

We derive a sample size calculation for a specific analysis: comparing the mean segmentation accuracy — i.e. the proportion of voxels in an image that match the reference standard L — of two algorithms A and B that generate binary classifications of *v* voxels on *n* images using a paired Student’s *t*-test (Rosner, 2015) on the per-image accuracies. Specifically, this tests the null hypothesis that the mean segmentation accuracies of A and B (both measured by comparison to L) are equal against the alternative hypothesis that they are unequal. Paired *t*-test analyses such as this one are frequently performed in comparisons of segmentation accuracy (Caballero et al., 2014).

### 2.1 Notation

Throughout this paper, we use the notation given in Table 1. Symbols used in this paper are summarized in Table 2.

**Table 1:** Notation for mathematical symbols.

Type	notation
Segmentation algorithms	X (upper case non-italic)
Random variables and vectors	*X* (upper case)
Realizations of random variables and constants	*x* (lower case)
Vectors	$\vec{x}$ (arrow accent); 〈 *x, y* 〉 (angle brackets)
Estimates	$\hat{x}$ (circumflex accent)
Parameterized distributions	*X* ∼ **X**(*θ*) (bold capital with parameters in parentheses)
Expectation of *X*	*E*[*X*]
Conditional expectation of *X* given *Z*	*E*(*X*|*Z*)
Conditional variance of *X* given *Z*	$\sigma_{X|Z}^{2}$
Conditional covariance of *X* and *Y* given *Z*	*cov*(*X, Y*|*Z*)
Event X=1	**x** (bold lower case)
Event X=0	$\bar{x}$ (bold lower case with bar)

**Table 2:** Glossary of mathematical symbols.

Symbol	Support	Description
Experimental parameters		
*n*	N	Sample size
*v*	N	Number of voxels per image
*α*	R	Significance threshold (acceptable Type I error)
*β*	R	1−power (acceptable Type II error)
*δ_MDD_*	[−1,1]	Minimum difference to detect with specified power
Population parameters		
$\vec{p}$	[0, 1]^3^	Population average marginal probability for the per-voxel accuracy difference
*δ*	[−1,1]	Population accuracy difference
*ψ*	[0, 1]	Probability that A and B disagree on voxel label
*δ_H_*	[−1,1]	Population accuracy difference measured against high-quality reference standard H
*p*(**a**), *p*(**b**), *p*(**l**), *p*(**h**)	[0, 1]	Probabilities of voxel labels being 1 for a randomly selected voxel
*ρ*_*i, j*_	[−1,1]	Correlation between *D*_*k, i*_ and *D*_*k, j*_ given ${\vec{O}}_{k}$
$\bar{\rho_{i,j}}$	[0, 1]	Average *ρ*_*i, j*_ over all voxel pairs *i* and *j*
$\sigma_{O_{1}-O_{-1}}^{2}$	$\left[0,\psi -\delta^{2}\right]$	Variance of the accuracy difference in the marginal probability prior
*ω*	$\omega \in R^{+}$	Precision parameter of Dirichlet distribution controlling inter-image variability
Random variables		
*A*_*k, i*_, *B*_*k, i*_, *L*_*k, i*_, *H*_*k, i*_	{0, 1}	Segmentation label for the *i*th voxel in the *k*th image
${\vec{O}}_{k}$	[0, 1]^3^	Per-image prior on average marginal probability
${\vec{O}}_{k,i}$	[0, 1]^3^	Per-voxel prior on marginal probability
${\vec{D}}_{k}$	${\left\{-1,0,1\right\}}^{v}$	Vector of per-voxel accuracies for the *k*th image
*D*_*k, i*_	{−1,0,1}	Difference in accuracy for the *i*th voxel of the *k*th image
*D*	{−1,0,1}	Difference in accuracy for a random voxel
${\bar{D}}_{k}$	[−1,1]	Per-image accuracy difference
Simulation variables		
*Dist*_*i, j*_	$R^{+}$	Distance between voxels *i* and *j*
*σ_ρ_*	$R^{+}$	Scaling parameter to control spatial correlation in Monte Carlo simulations
${\bar{d}}_{k}$	[−1,1]	Per-image accuracy difference of a simulated image
*d*_*k, i*_	{−1,0,1}	Per-voxel accuracy difference of a simulated voxel
Other notation		
$p_{-1},$*p*_0_, *p*_1_	[0, 1]	Elements of $\vec{p}$ for values −1, 0, and 1
$O_{k,-1},$*O*_*k*, 0_, *O*_*k*, 1_	[0, 1]	Elements of ${\vec{O}}_{k}$ for values −1, 0, and 1
$O_{k,i,-1},$*O*_*k, i*, 0_, *O*_*k, i*, 1_	[0, 1]	Elements of ${\vec{O}}_{k,i}$ for values −1, 0, and 1
A, B, L, H		Segmentation sources denoting two algorithms, a low-quality and a high-quality reference
*f*		Design factor
*t*_*p*{1}_, *t*_*p*{2}_	R	1- and 2-tailed *p* probability critical value from a **T**-distribution
$\sigma_{0}^{2}$	$\left[0,\sqrt{2}\right]$	Per-image accuracy difference variance under the null hypothesis
$\sigma_{alt}^{2}$	$\left[0,\sqrt{2}\right]$	Per-image accuracy difference variance under the alternative hypothesis

[*x, y*] denotes real numbers between *x* and *y*; {*x, y, z*} denotes a set of possible values; a superscript ^*x*^ denotes a vector with *x* elements; N denotes natural numbers; R denotes real numbers. $R^{+}$ denotes positive real numbers.

### 2.2 Statistical model of segmentation

Our stochastic population model represents the joint distribution of possible segmentations by A, B, and L over a population of images. The data for one image from this population comprises binary segmentation labels (encoded as integers 0 or 1) assigned by A, B and L to each of the *v* voxels: $a_{k,1},\ldots ,a_{k,v},b_{k,1},\ldots ,b_{k,v},l_{k,1},\ldots ,l_{k,v},$ where *a*_*k, i*_, *b*_*k, i*_, and *l*_*k, i*_ are the labels for the *i*th voxel in the *k*th image. The data for a study comprises *n* randomly sampled images, which we denoted with a set of random variables $\{A_{k,1},\ldots ,A_{k,v},B_{k,1},\ldots ,B_{k,v},L_{k,1},\ldots ,L_{k,v}|k=1..n\},$ where *A*_*k, i*_, *B*_*k, i*_, and *L*_*k, i*_ are the random variables representing labels for the *i*th voxel in the *k*th randomly sampled image.

#### 2.2.1 Accuracy difference measures

We focus on three types of segmentation accuracy differences. First, the *per-voxel segmentation accuracy difference* for the *i*th voxel in the *k*th image is $D_{k,i}=|B_{k,i}-L_{k,i}\left|-\right|A_{k,i}-L_{k,i}|$. *D*_*k, i*_ can take on three values: 1 (when $A_{k,i}=L_{k,i}\neq B_{k,i}$), 0 (when $A_{k,i}=B_{k,i}$) and −1 (when $A_{k,i}\neq L_{k,i}=B_{k,i}$). Random vector ${\vec{D}}_{k}$ represents all *D*_*k, i*_ for the *k*th image. Second, the *per-image accuracy difference* is the proportion of correct voxel labels from algorithm A (with respect to reference standard L) minus the proportion of correct voxel labels from algorithm B (with respect to reference standard L): ${\bar{D}}_{k}=\frac{1}{v}\sum_{i=1}^{v}\left(1-\right|A_{k,i}-L_{k,i}\left|\right)-\frac{1}{v}\sum_{i=1}^{v}\left(1-\right|B_{k,i}-L_{k,i}\left|\right)=\frac{1}{v}\sum_{i=1}^{v}D_{k,i}$. Third, the population average accuracy difference *δ* is the expected value $E[{\bar{D}}_{k}]$ for a randomly selected image in the population, and equivalently, δ=p(D=1)−p(D=−1) for a randomly selected per-voxel accuracy difference *D*.

#### 2.2.2 Model distribution

**Fig. 2:**
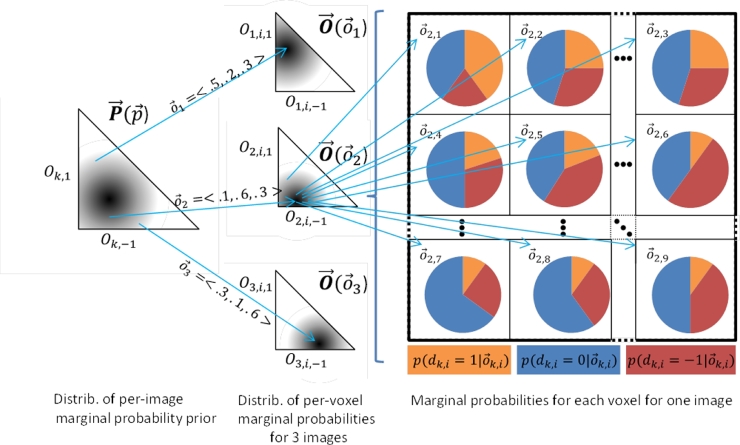
The illustrated nested model shows, from left to right, (1) the prior distribution of per-image average marginal probabilities $\vec{P}\left(\vec{p}\right)$ (shown on the triangular (standard 2-simplex) domain with axes *O*_*k*, 1_ and $O_{k,-1}$ shown and *O*_*k*, 0_ implicitly defined as $1-O_{k,1}-O_{k,-1}$; darkness represents the probability density), (2) three different samples (i.e. three images) of per-image average marginal probabilities ${\vec{o}}_{k}$ (shown as arrows labelled ${\vec{o}}_{1},$${\vec{o}}_{2}$ and ${\vec{o}}_{3}$), (3) three corresponding conditional prior distributions of per-voxel marginal probabilities $\vec{O}\left({\vec{O}}_{k}\right)$ for the three images (shown as in (1)), (4) nine different samples (i.e. nine voxels from the second image) of per-voxel marginal probabilities ${\vec{o}}_{k,i}$ (shown as unlabelled arrows), and (5) the categorical distributions for the nine voxels from the second image (shown as pie charts of the relative probabilities of the per-voxel accuracy differences $p(d_{k,i}=1|{\vec{o}}_{k,i})$ [orange], $p(d_{k,i}=0|{\vec{o}}_{k,i})$ [blue], and $p(d_{k,i}=-1|{\vec{o}}_{k,i})$ [red]). (For interpretation of the references to colour in this figure legend, the reader is referred to the web version of this article.)

For calculating power, the model (summarized in Table 3 and illustrated in Fig. 2) must encode the distribution of the metric analysed in the statistical analysis: the per-image accuracy difference ${\bar{D}}_{k}$. While ${\bar{D}}_{k}$ depends on all three segmentations A, B and L, it can be expressed more simply as a unary function of ${\vec{D}}_{k}$. Therefore, we consider the distribution of ${\vec{D}}_{k}$ directly, modeled as a *v*-dimensional correlated categorical distribution. To model this distribution, we follow the common convention of breaking down complex joint distributions into the mean, and multiple simpler sources of variation about the mean.

**Table 3:** Model summary. These expressions summarize the nested model used in our derivations. The motivation and detailed description is given in Section 2.2.2.

${\vec{O}}_{k}∼\vec{P}\left(\vec{p}\right)$ where $E\left[{\vec{O}}_{k}\right]=\vec{p}$	
$\forall_{i}{\vec{O}}_{k,i}∼\vec{O}\left({\vec{O}}_{k}\right)$ where $E\left({\vec{O}}_{k,i}|{\vec{O}}_{k}\right)={\vec{O}}_{k}$	
$\forall_{i}D_{k,i}∼\mathrm{Categorical}\left({\vec{O}}_{k,i}\right)$	
$\forall_{i\neq j}cov\left(D_{k,i},D_{k,j}|{\vec{O}}_{k}\right)=\rho_{i,j}\sqrt{\sigma_{D_{k,i}|{\vec{O}}_{k}}^{2}\sigma_{D_{k,j}|{\vec{O}}_{k}}^{2}}$	

The mean of ${\vec{D}}_{k}$ is defined by the joint distribution of the segmentation labels. Considering the joint distribution is important, because the algorithm and reference standard labels for a randomly selected voxel (*A, B* and *L*) may not be independent from each other, as they depend on the same image information and overlapping prior knowledge. The mean of ${\vec{D}}_{k},$ therefore, encodes the inter-segmentation correlation in the population average marginal probabilities of the per-voxel accuracy difference *D* (marginalized over combinations of segmentations A, B and L yielding each difference value):

(1) $$\begin{array}{ccc} & & p(D=1)=p(A=1,B=0,L=1)+p(A=0,B=1,L=0);\\ & & p(D=0)=p(A=B);\\ & & p(D=-1)=p(A=1,B=0,L=0)\;+\;p(A=0,B=1,L=1). \end{array}$$

For example, when *A* and *B* are highly correlated, p(D=0) is higher and when *A* and *L* are highly correlated, p(D=1) increases while p(D=−1) decreases. We consider the population average marginal probabilities as a model parameter $\vec{p}=<p_{1},p_{0},p_{-1}>=<p\left(D=1\right),p\left(D=0\right),p\left(D=-1\right)>$.

The variation of ${\vec{D}}_{k}$ about the mean is affected by three sources of variation:

• **intra-image inter-voxel correlation** – two voxels in the same image may have correlated labels if, for example, they are adjacent or are commonly affected by the same image artifact.
• **inter-image variability** – the expected segmentation performance for different images may vary, as one image may have features that are more or less challenging for a particular algorithm or observer than another image.
• **inter-voxel variability** – two voxels in the same image may have different marginal probabilities depending on the image content; for example, voxels that are easy to segment for any algorithm would likely have the same labels for any algorithm, where more challenging voxels are more likely to show differences.

Both the inter-image variability and the intra-image inter-voxel correlation affect the covariance matrix of ${\vec{D}}_{k}$. While the covariance matrix could be an explicit model parameter, interpreting the parameter is challenging because it conflates these different sources of correlation. Instead, we construct an over-parameterized nested model that allows us to separately represent inter-image variability and intra-image inter-voxel correlation. The key concept in this nested model is to introduce per-image priors (random variables ${\vec{O}}_{k}∼\vec{P}\left(\vec{p}\right)$) on the average marginal probability for *D*_*k, i*_ within each image, in order to model inter-image variability. $\vec{P}\left(\vec{p}\right)$ is a distribution of probability vectors (i.e. ${\vec{O}}_{k}\in$ the open standard 2-simplex) with mean $\vec{p}$. Then, for each image, the conditional distribution of *D*_*k, i*_ given ${\vec{O}}_{k}$ models the intra-image inter-voxel correlation. Specifically, we define the conditional covariance of ${\vec{D}}_{k}$ given ${\vec{O}}_{k}$ as

(2) $$\begin{array}{c} cov\left(D_{k,i},D_{k,j}|{\vec{O}}_{k}\right)=\rho_{i,j}\sqrt{\sigma_{D_{k,i}|{\vec{O}}_{k}}^{2}\sigma_{D_{k,j}|{\vec{O}}_{k}}^{2}}, \end{array}$$

where *ρ*_*i, j*_ is a pair-wise Pearson correlation coefficient and $\sigma_{D_{k,i}|{\vec{O}}_{k}}^{2}$ is the conditional variance of *D*_*k, i*_ given ${\vec{O}}_{k}$.

To model the inter-voxel variability, each *D*_*k, i*_ has per-voxel priors (random variables ${\vec{O}}_{k,i}$) defining its marginal probabilities. The conditional distribution of ${\vec{O}}_{k,i}$ given ${\vec{O}}_{k}$ is an arbitrary distribution $\vec{O}\left({\vec{O}}_{k}\right)$ of probability vectors with mean ${\vec{O}}_{k}$.

### 2.3 Derivation of the sample size formula for segmentation

The general form of the sample size formula (Connor, 1987),

(3) $$\begin{array}{c} n=\frac{{\left(t_{\alpha \{2\}}\sqrt{\sigma_{0}^{2}}+t_{\beta \{1\}}\sqrt{\sigma_{alt}^{2}}\right)}^{2}}{\delta_{MDD}^{2}}, \end{array}$$

relates the sample size (*n*) to the variances ($\sigma_{0}^{2}$ and $\sigma_{alt}^{2}$) of per-image accuracy differences under the null hypothesis (δ=0) and alternate hypothesis (*δ* ≠ 0), acceptable study error rates (*α* and *β*), and the minimum detectable difference (*δ_MDD_*) in population accuracy between algorithms A and B to detect with power (1−β). *t*_*α*{2}_ and *t*_*β*{1}_ are two- and one-tailed critical values taken from the inverse cumulative distribution function of the *t*-distribution with n−1 degrees of freedom. Of the parameters in Eq. (3), most are selected based on experimental design choices, but the variances of the per-image accuracy difference are derived from the statistical model.

**Fig. 3:**
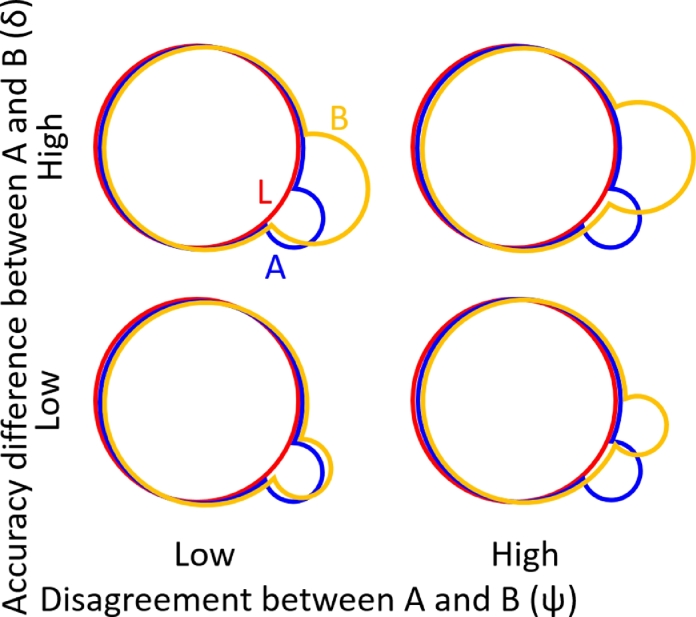
Illustration of the relationship between the proportion of disagreement (*ψ*) and the accuracy difference (*δ*). In these four examples, segmentation algorithms A (blue) and B (yellow) both over-contour the circular object taken as the reference standard segmentation L (red), adding different perturbations that lower accuracy. When sets of segmentations have higher *ψ* and lower *δ* (as in the lower right), it is harder to detect accuracy differences. (For interpretation of the references to colour in this figure legend, the reader is referred to the web version of this article.)

The variance of the per-image accuracy difference $\sigma_{\bar{D}}^{2}$ can be derived for any prior distribution of per-image average marginal probabilities (${\vec{O}}_{k}∼\vec{P}\left(\vec{p}\right)$) in terms of moments of the prior distribution by marginalizing out ${\vec{O}}_{k}$ and ${\vec{O}}_{k,i}$ (see Appendix A for a detailed derivation), yielding

(4) $$\begin{array}{c} \sigma_{\bar{D}}^{2}=\bar{\rho_{i,j}}\left(\psi -\delta^{2}\right)+\left(1-\bar{\rho_{i,j}}\right)\sigma_{O_{1}-O_{-1}}^{2}, \end{array}$$

where $\psi =p_{1}+p_{-1}$ is the population-wide probability that algorithms A and B disagree on the labeling of a voxel (see Fig. 3), $\sigma_{O_{1}-O_{-1}}^{2}$ (the variance of $O_{k,1}-O_{k,-1}$ for the priors ${\vec{O}}_{k}$) is a linear combination of moments of the prior distribution ($\sigma_{O_{1}-O_{-1}}^{2}=\sigma_{O_{1}}^{2}-2\sigma_{O_{1},O_{-1}}+\sigma_{O_{-1}}^{2}$), and $\bar{\rho_{i,j}}=\frac{\sum_{i,j}\rho_{i,j}}{v^{2}}$ is the average of the intra-image inter-voxel correlation coefficients.

Substituting $\sigma_{alt}^{2}=\sigma_{\bar{D}|\delta =\delta_{MDD}}^{2}$ and $\sigma_{0}^{2}=\sigma_{\bar{D}|\delta =0}^{2}$ (i.e. substituting $\delta =\delta_{MDD}$ and δ=0 into $\sigma_{\bar{D}}^{2}$) yields the segmentation sample size formula for accuracy differences with respect to reference standard L,

(5) $$\begin{array}{ccc} n & = & (t_{\alpha \{2\}}\sqrt{\bar{\rho_{i,j}}\psi +\left(1-\bar{\rho_{i,j}}\right)\sigma_{O_{1}-O_{-1}|\delta =0}^{2}}\\ & & +{t_{\beta \{1\}}\sqrt{\bar{\rho_{i,j}}\left(\psi -\delta_{MDD}^{2}\right)+\left(1-\bar{\rho_{i,j}}\right)\sigma_{O_{1}-O_{-1}|\delta =\delta_{MDD}}^{2}})}^{2}/\delta_{MDD}^{2}. \end{array}$$

It is interesting to note that when there is no inter-voxel correlation (i.e. $\bar{\rho_{i,j}}\to 1/v$) and no inter-image variability in marginal probabilities (i.e. $\sigma_{O_{1}-O_{-1}}^{2}=0$), Eq. (5) approaches the sample size formula for McNemar’s two-sample paired proportion test with *nv* samples (Connor, 1987).

#### 2.3.1 Sample size with the Dirichlet prior distribution

To gain further insight into the sample size relationship, consider the special case where the prior distribution of per-image average marginal probabilities $\vec{P}\left(\vec{p}\right)$ is a Dirichlet distribution (i.e. ${\vec{O}}_{k}∼\mathrm{Dirichlet}\left(\omega ,\vec{p}\right)$), which represents inter-image variability with a single parameter: the precision *ω* (Minka, 2000). When *ω* is large, priors ${\vec{O}}_{k}$ are likely to be near $\vec{p}$ (i.e. there is little variation between images); when *ω* is small, priors ${\vec{O}}_{k}$ are distributed more diffusely (i.e. there is more variation between images). The Dirichlet prior distribution has three properties that make interpretation of the sample size relationship easier:

• It is well-characterised as a model for variability in categorical probabilities, because it is the conjugate prior distribution of the categorical and multinomial distributions and thus commonly adopted in Bayesian analysis (Tu, Mosimann, 1962, Zhu, Zöllei, Wells, 2006)
• Representing inter-image variability with a single parameter simplifies interpretation and facilitates parameter fitting with small pilot data sets.
• $\sigma_{O_{1}-O_{-1}}^{2}$ for the Dirichlet prior distribution is proportional to $\psi -\delta^{2}$ which simplifies the sample size formula.

For the Dirichlet prior distribution, $\sigma_{O_{1}}^{2}=\frac{p_{1}-p_{1}^{2}}{\omega +1},$$\sigma_{O_{1},O_{-1}}=\frac{-p_{1}p_{-1}}{\omega +1},$ and $\sigma_{O_{-1}}^{2}=\frac{p_{-1}-p_{-1}^{2}}{\omega +1}$; therefore $\sigma_{O_{1}-O_{-1}}^{2}=\frac{\psi -\delta^{2}}{\omega +1}$. Substituting $\sigma_{O_{1}-O_{-1}}^{2}$ into Eq. (4) and simplifying algebraically gives the variance of the per-image accuracy under a Dirichlet prior:

(6) $$\begin{array}{c} \sigma_{\bar{D}}^{2}=\frac{1+\omega \bar{\rho_{i,j}}}{\omega +1}\left(\psi -\delta^{2}\right). \end{array}$$

Since $\sigma_{\bar{D}}^{2}$ is expressed in terms of *δ*, we can readily substitute $\sigma_{alt}^{2}=\sigma_{\bar{D}|\delta =\delta_{MDD}}^{2}$ and $\sigma_{0}^{2}=\sigma_{\bar{D}|\delta =0}^{2}$ into Eq. (3) to get the sample size formula

(7) $$\begin{array}{c} n=\frac{1+\omega \bar{\rho_{i,j}}}{\omega +1}{\left(t_{\alpha /2}\sqrt{\psi /\delta_{MDD}^{2}}+t_{\beta }\sqrt{\psi /\delta_{MDD}^{2}-1}\right)}^{2}. \end{array}$$

Several aspects of this formula link to previous work. The term $\frac{1+\omega \bar{\rho_{i,j}}}{\omega +1}$ is a type of *design factor* denoted hereafter as *f* (analogous to the design factor in cluster-randomized trials (Kish, 1965)), modelling the inter- vs intra-image variability in accuracy differences (i.e. each image being one correlated cluster of voxel samples). When there is no inter-voxel correlation (i.e. $\bar{\rho_{i,j}}=1/v$), Eq. (7) simplifies to the formula found in our preliminary analysis (Gibson et al., 2015). The term *ψ*/*δ*^2^ is the squared coefficient of variation of *D* under the idealized assumption of completely independent voxels (i.e. f=1/v) — or equivalently, the statistical efficiency of estimating *δ* (Everitt and Skrondal, 2002). We thus refer to *ψ*/*δ*^2^ hereafter as the *idealized efficiency*.

### 2.4 Incorporating reference standard quality

Conducting segmentation accuracy comparison studies using a lower-quality reference standard introduces an additional challenge: selecting the appropriate minimum detectable difference. On one hand, for the generic sample size formula (Eq. (3)) to be valid, *δ_MDD_* must be measured with respect to the reference standard used in the study. On the other hand, the selection of *δ_MDD_* depends on external clinical or technical requirements. Ideally, these requirements would be defined with respect to a high-quality reference standard H (with the MDD denoted *δ*_*MDD, H*_), to most closely approximate the true requirement. If the high-quality reference standard can be used for the entire study, there is no conflict and *δ*_*MDD, H*_ can be used directly. If, however, a lower-quality reference standard is used, an appropriate *δ_MDD_* needs to be selected. To resolve this dilemma, we have derived a formula to express *δ_MDD_* for a low-quality reference standard as a function of *δ*_*MDD, H*_, by characterizing the differences between the low- and high-quality reference standards (e.g. on a small pilot dataset).

The derivation, detailed in Appendix B, expresses *δ_MDD_* in terms of the joint probability of segmentation labels of A, B, L and H; isolates the terms of this expression that equate to *δ*_*MDD, H*_; and simplifies the remaining terms. This yields an equation for *δ_MDD_* as a function of *δ*_*MDD, H*_ and estimable parameters representing deviation of *δ_MDD_* from *δ*_*MDD, H*_:

(8) $$\begin{array}{ccc} \delta_{MDD} & = & \delta_{MDD,H}+2\left(p\left(a\right)-p\left(b\right)\right)\left(p\left(l\right)-p\left(h\right)\right)\\ & & +\;2cov(A-B,L-H), \end{array}$$

where p(x)=p(X=1) for a randomly selected voxel and cov(A−B,L−H) is the covariance between errors in L (with respect to H) and differences between A and B. The second term of this expression reflects error induced by over- or under-contouring by L (with respect to H). If L tends to over-contour compared to H, algorithms that assign more voxels as foreground will appear more accurate. The third term is the covariance cov(A−B,L−H) reflecting errors in L that are biased in favour of A or B. This expression can be used to estimate the *δ_MDD_* to use for a study using a low-quality reference standard.

## 3 Applying the sample size formula

The sample size formula derived above supports the design of segmentation accuracy comparison studies by estimating the sample size needed to detect a specified accuracy difference with high probability. As with all sample size calculations, three types of parameters have to be determined to apply the formula: the acceptable study error rates, the minimum detectable difference, and the variance parameters. Some of these parameters are chosen based on experimental, technical or clinical requirements outside the study design, while others are estimated from related literature or pilot data. We denote the estimate of parameter *x* as $\hat{x}$.

The acceptable error rates are generally set using heuristics by study designers: α=0.05 (i.e. a 5% probability of falsely detecting a difference when there is none) and β=0.2 (i.e. an 80% probability of detecting a true difference).

The minimum detectable difference (*δ_MDD_*) is typically set by technical or clinical requirements *outside the study design* to be the smallest difference that is large enough to be important to detect with high probability. Specifically, if the true difference is *δ_MDD_* or higher, the study should give a true positive with probability 1−β or higher. If the study will use a sufficiently high-quality reference standard, *δ_MDD_* can be chosen directly. If the technical or clinical requirements are expressed with respect to a high-quality reference standard, but the study uses a lower-quality reference standard, then *δ*_*MDD, H*_ can be chosen and the equivalent ${\hat{\delta }}_{MDD}$ can be estimated from the low-quality correction equation (Eq. (8)), using parameter estimation equations (Eqs. (9) and (10)) given in Section 3.1.

The variance parameters depend on the distribution of the data; they are not chosen a priori, but can be estimated using values from related literature, or using pilot data. In the moment-based sample size equation (Eq. (5)), the variance parameters are *ψ*, $\bar{\rho_{i,j}},$$\sigma_{O_{1}-O_{-1}|\delta =0}^{2}$ and $\sigma_{O_{1}-O_{-1}|\delta =\delta_{MDD}}^{2}$. In the Dirichlet-prior-based sample size equation (Eq. (7)), the variance parameters are *ψ*, $\bar{\rho_{i,j}},$ and *ω*. In general, estimating these variance parameters individually can be challenging because the model is parameterized by multiple parameters that affect the intervoxel covariance of per-voxel accuracy differences, and because the moments of the prior for the per-image average marginal probabilities may depend on *δ*. Under some assumptions, however, we can estimate variance parameters.

• If we assume $\sigma_{0}^{2}=\sigma_{alt}^{2}={\hat{\sigma }}_{\bar{D}}^{2},$ which may be appropriate when *δ* and *δ_MDD_* are sufficiently small, we can estimate ${\hat{\sigma }}_{\bar{D}}^{2}$ from the pilot data (using Eq. (13) in Section 3.1), and apply the generic sample size equation (Eq. (3)) directly.
• If we assume a parametric distribution for the per-image average marginal probabilities, it may be possible to express $\sigma_{O_{1}-O_{-1}}^{2}$ in terms of *δ* (as shown for the Dirichlet distribution in Eq. (6)) and estimate $\sigma_{O_{1}-O_{-1}|\delta =0}^{2}$ and $\sigma_{O_{1}-O_{-1}|\delta =\delta_{MDD}}^{2}$ from ${\hat{\sigma }}_{\bar{D}}^{2}$. For the Dirichlet distribution, the resulting variance could be characterized by a design factor modeling the combined effect of parameters $\bar{\rho_{i,j}},$ and *ω*. An estimation equation for the design factor is given in Section 3.1 Eq. (14).
• If there is a need to estimate the effects of the variance parameters individually (e.g. to explore the effect of increased intra-image inter-voxel correlation on a planned study), and we assume that the intra-image inter-voxel correlation is spatially constrained (e.g. if voxels separated by a specified distance are effectively uncorrelated given ${\vec{O}}_{k}$), then we can estimate $\hat{\omega }$ using spatially sparse sampling and then estimate $\hat{\bar{\rho_{i,j}}}$ from $\hat{\omega }$ and ${\hat{\sigma }}_{\bar{D}}^{2}$. This approach is outlined for a Dirichlet prior in Section 3.1.

The optimal size for a pilot study data set has not been well-established in general, and depends on many factors (Hertzog, 2008), including the particular population being studied. In principle, the precision of the estimated sample size depends on the sensitivity of the formula to parameter estimation errors (see supplementary material) and the variances of the parameter estimators (which decrease as the pilot data set grows), both of which vary depending on the population being studied. In practice, formal sample size calculations for such pilot studies are rarely used (Hertzog, 2008); instead, heuristics, such as using 10 samples (Nieswiadomy, 2011), 12 samples (Julious, 2005) or using 10% of the anticipated size of the full study (Connelly, 2008, Lackey, Wingate, 1986) for larger studies, can be used. The risk of parameter estimation error can be mitigated using conservative parameter estimates, as described in Section 3.1 for ${\hat{\sigma }}_{\bar{D}}^{2}$.

### 3.1 Parameter estimation equations

To estimate parameters from pilot data, a small data set of images must be collected and segmented by algorithms A and B, by the reference standard L to be used for the study, and by the high-quality reference standard H. Given a segmented pilot data set, formula parameters can be estimated as follows.

To estimate ${\hat{\delta }}_{MDD}$ in terms of *δ*_*MDD, H*_, we first estimate the proportion of positive voxels segmented by A across all images in the pilot data:

(9) $$\begin{array}{c} \hat{p}\left(a\right)=\frac{1}{n^{\prime }v}\sum_{k=1}^{n^{\prime }}\sum_{i=1}^{v}a_{k,i}, \end{array}$$

where *n*′ is the number of images in the pilot data set. $\hat{p}\left(b\right),$$\hat{p}\left(l\right),$ and $\hat{p}\left(h\right)$ can be estimated similarly. $\hat{cov}\left(A-B,L-H\right)$ can be estimated as

(10) $$\begin{array}{ccc} \hat{cov}\left(A-B,L-H\right) & = & \frac{1}{n^{\prime }v-1}\sum_{k=1}^{n^{\prime }}\sum_{i=1}^{v}(a_{k,i}-b_{k,i}-\hat{p}\left(a\right)\\ & & +\;\hat{p}\left(b\right))\left(l_{k,i}-h_{k,i}-\hat{p}\left(l\right)+\hat{p}\left(h\right)\right). \end{array}$$

Then, from Eq. (8), ${\hat{\delta }}_{MDD}=\delta_{MDD,H}+2\left(\hat{p}\left(a\right)-\hat{p}\left(b\right)\right)\left(\hat{p}\left(l\right)-\hat{p}\left(h\right)\right)+2\hat{cov}\left(A-B,L-H\right)$.

The probability of disagreement can be estimated using the sample mean as

(11) $$\begin{array}{c} \hat{\psi }=\frac{1}{n^{\prime }v}\sum_{k=1}^{n^{\prime }}\sum_{i=1}^{v}\left|a_{k,i}-b_{k,i}\right|. \end{array}$$

The population average accuracy difference can be estimated using the sample mean as

(12) $$\begin{array}{c} \hat{\delta }=\frac{1}{n^{\prime }v}\sum_{k=1}^{n^{\prime }}\sum_{i=1}^{v}\left(|b_{k,i}-l_{k,i}\left|-\right|a_{k,i}-l_{k,i}|\right). \end{array}$$

The variance in per-image accuracy differences can be estimated using the unbiased sample variance as

(13) $$\begin{array}{c} {\hat{\sigma }}_{\bar{D}}^{2}=\frac{1}{\left(n^{\prime }-1\right)}\sum_{k=1}^{n^{\prime }}{\left({\bar{d}}_{k}-\hat{\delta }\right)}^{2}, \end{array}$$

where ${\bar{d}}_{k}=\frac{1}{v}\sum_{i=1}^{v}\left(\right|b_{k,i}-l_{k,i}\left|-\right|a_{k,i}-l_{k,i}\left|\right)$. However, sample variance estimates from small pilot studies are imprecise and skewed (Browne, 1995), which inflates the probability of having an underpowered study. To mitigate this effect, Browne (1995) recommended using the upper bound of a *γ*% confidence interval on the variance to guarantee the specified power with *γ*% probability. This can be estimated using a double bootstrap method (e.g. Lee and Young, 1995 implemented for Matlab as *ibootci* (Penn, 2015)).

When modeling the per-image marginal probability prior as a Dirichlet distribution, the design factor encoding the combined effect of parameters $\bar{\rho_{i,j}},$ and *ω* can be estimated from Eq. (6) using sample estimates:

(14) $$\begin{array}{c} \hat{f}={\hat{\sigma }}_{\bar{D}}^{2}/\left(\hat{\psi }-{\hat{\delta }}^{2}\right), \end{array}$$

and the idealized efficiency can be estimated as $\hat{\psi }/\delta_{MDD}^{2}$.

To estimate the effects of the variance parameters individually, we can model the per-image marginal probability prior as a Dirichlet distribution and assume that the intra-image inter-voxel correlation is spatially constrained (i.e. voxels more than *x* pixels away are effectively uncorrelated given ${\vec{O}}_{k}$). Sampling *d*_*k, i*_ from voxels spaced *x* voxels apart gives counts from a Dirichlet-multinomial distribution, and we can estimate the precision parameter $\hat{\omega }$ using an iterative approach described by Minka (2000). The average correlation coefficient can then be estimated from Eq. (6) using sample estimates as

(15) $$\begin{array}{c} \hat{\bar{\rho_{i,j}}}=\frac{{\hat{\sigma }}_{\bar{D}}^{2}\left(\hat{\omega }+1\right)-\left(\hat{\psi }-{\hat{\delta }}^{2}\right)}{\left(\hat{\psi }-{\hat{\delta }}^{2}\right)\hat{\omega }}. \end{array}$$

## 4 Simulations

Three sets of Monte Carlo simulations were used to evaluate the accuracy of the sample size formulae under three different conditions:

1. with simulated images and segmentations from the assumed statistical model, to test the validity of the model;
2. with real-world data (the PROMISE12 prostate MRI segmentation data set described in Section 4.2.1) using a high-quality reference standard, to test the applicability of the Dirichlet-based sample size formula (Eq. (7)) to real data; and
3. with real-world data using a low-quality reference standard while expressing the minimum detectable difference in terms of a high-quality reference standard, to test the applicability of the low-quality correction equation (Eq. (8)) to real data.

### 4.1 Simulations with simulated data from the assumed statistical model

In order to characterize the validity of the model described in Section 2.2, we performed sets of simulations with controlled variation of a subset of model parameters (hereafter referred to as a simulation set). Recall that Eq. (7) defines the sample size needed to detect a significant accuracy difference with probability 1−β *if the underlying population difference were δ_MDD_*. To test this, we set *δ_MDD_* to the specified population accuracy difference, and compare the proportion of simulated studies yielding significant accuracy differences to 1−β. Note that this approach to select *δ_MDD_* is appropriate for validating the sample size formula, but not for designing real segmentation comparison studies: in practice, *δ_MDD_* should be chosen based on clinical or technical requirements.

In each simulation, we repeatedly simulated a segmentation evaluation study by sampling per-voxel accuracy differences for ⌈*n*⌉ *v*-voxel segmentations and reference standards (where ⌈*n*⌉ denotes the smallest integer  ≥ *n*) using the assumed model and testing for an accuracy difference using a Student’s *t*-test. In each simulation, we compared the observed proportion of positive statistical tests with the predicted probability (i.e. the statistical power 1−β) for sample size ⌈*n*⌉. To clarify the impact of this error in power, we also substituted the observed power into the Dirichlet-based sample size formula (Eq. (7)) to calculate the equivalent error in the predicted sample size *n* and detectable difference *δ_MDD_*. In each simulation, we ran 25,000 repetitions in order to estimate the probability of a positive outcome with a 95% confidence interval with a width of 1%.

Each per-image accuracy difference $\bar{d}$ was computed by sampling the derived per-voxel accuracy differences *d*_*k, i*_ directly as follows:

• the marginal probability priors of per-voxel accuracy differences were drawn from a Dirichlet prior using the *rdirichlet* (Warnes et al., 2015) function in R version 3.1.1 (R Core Team, 2013),
• a correlation matrix $\rho_{i,j}=exp\left(-Dist_{i,j}/\sigma_{\rho }^{2}\right)$ was constructed where *Dist*_*i, j*_ is the intervoxel distance in a $\sqrt{v}\times \sqrt{v}$ voxel image and $\sigma_{\rho }^{2}$ is a scale parameter controlling the spatial extent of the correlation
• *d*_*k, i*_ were sampled using the *ordsample* (Barbiero and Ferrari, 2015) function in R. While this is equivalent to drawing samples from the algorithm and reference standard segmentations and computing *d*_*k, i*_, it facilitates the direct control of the *d*_*k, i*_ correlation matrix needed in these experiments.

The scripts used to generate these samples are available at https://github.com/eligibson/MedIA2016.

The baseline parameter values in the simulation sets and the ranges of varied parameters are given in Table 4. Note that the simulations varying *v, ω, σ_ρ_* and *ψ* were conducted at two baseline *δ* values. The parameter ranges for these simulations were chosen to balance the applicability of parameter values to medical image segmentation problems against practical constraints. The range of *ω* encompassed both highly consistent and highly variable prior distributions. Ranges of *δ* and *ψ* reflected plausible algorithm differences based on previous experience. Due to limitations on the *ordsample* algorithm the range of *v* and *σ_ρ_* were constrained: *v* was limited to 100 because of the computational complexity of sampling high-dimensional correlated discrete random variables, and *σ_ρ_* was constrained to 0.7 because of algorithmic constraints. The baseline parameter values were chosen to reflect typical sample sizes in segmentation studies (∼10−∼200). Because the population parameters derived in Section 2.4 (*δ_H_, p*(**a**), *p*(**b**), *p*(**l**), *p*(**h**) and cov(A−B,L−H)) are linked to statistical power through their influence on the parameter *δ*, simulations were run as a function of *δ*, instead of simulating many combinations of parameters that map to the same *δ*.

**Table 4:** Simulation parameters used to estimate the accuracy of the model. Note that the simulations varying *v, ω, σ_ρ_* and *ψ* were conducted twice at two baseline *δ* values.

	# voxels	population accuracy difference	Dirichlet precision	spatial correlation width	population probability of disagreement
	*v*	*δ*	*ω*	*σ_ρ_*	*ψ*
Baseline	36	3% / 6%	128	0.7	15%
Minimum	9	2%	64	0	15%
Maximum	100	10%	1024	0.7	45%
Increment	$\sqrt{v}$ by +1	+1%	× 2	+0.1	+5%

### 4.2 Simulations with real-world data

To evaluate the applicability of sample size formula (Eq. (7)) and the low-quality correction equation (Eq. (8)) to a real-world data set, we simulated segmentation accuracy comparison studies using bootstrapped samples from the PROMISE12 data set.

The PROMISE12 challenge is an ongoing resource for comparing many state-of-the-art prostate segmentation algorithms against a common reference standard. The challenge images comprise 100 T2W prostate MR images collected from 4 centres, split into 50 training images (with publicly available reference segmentations) and 30 testing images (with reference segmentations withheld). The reference segmentations were manually segmented by an experienced clinical reader, and verified by another independent clinical reader. In order to establish a standardised scoring system for multiple metrics, the challenge had a non-clinical graduate student manually segment the images and her metric scores were used to normalize the metric scores of the algorithms. Although the PROMISE12 challenge principally used the high-quality reference standard for evaluation, the second segmentation is analogous to a presumably lower-quality reference standard that could be considered as a lower cost option. Thus, the clinical manual segmentations will represent the high-quality reference standard H, the graduate student manual segmentations will represent the low-quality reference standard L, and two algorithms from the challenge will represent A and B. Using 10 algorithms from the PROMISE12 challenge, the simulations were repeated for all 45 possible pairs of algorithms.

As in Section 4.1, we set *δ_MDD_* to the population accuracy difference (treating the PROMISE12 test data set as the entire population) and compare the proportion of simulated studies yielding significant accuracy differences to 1−β.

#### 4.2.1 Simulations with high-quality real-world data

To evaluate the applicability of the Dirichlet-based sample size formula (Eq. (7)) to a real-world data set, each simulated study in this experiment compared two algorithms to the high-quality reference standard. For every pair of algorithms, we estimated the population accuracy difference (${\hat{\delta }}_{H}$) and variance parameters using all 30 test cases from the PROMISE12 test data set. Using α=0.05,β=0.20,$\delta_{MDD}={\hat{\delta }}_{H},$ and the estimated variance parameters, we computed the predicted sample size *n* using Eq. (7). We then simulated 100,000 segmentation accuracy comparison studies using bootstrap sampling by sampling ⌈*n*⌉ images with replacement from the PROMISE12 images and testing the per-image accuracy differences using a paired Student’s *t*-test. We compared the proportion of positive tests to the power predicted by the model for ⌈*n*⌉ samples.

#### 4.2.2 Simulations with low-quality real-world data

To evaluate the applicability of the low-quality correction equation (Eq. (8)) to a real-world data set, each simulated study in this experiment compared two algorithms to the low-quality reference standard, with ${\hat{\delta }}_{MDD}$ calculated from Eq. (8) and the observed ${\hat{\delta }}_{H}$. Simulation using bootstrap sampling and evaluation proceeded as in Section 4.2.1 except that ${\hat{\delta }}_{MDD}={\hat{\delta }}_{H}+2\left(\hat{p}\left(a\right)-\hat{p}\left(b\right)\right)\left(\hat{p}\left(l\right)-\hat{p}\left(h\right)\right)+2\hat{cov}\left(A-B,L-H\right),$ and the variance parameters were estimated with respect to low-quality reference standard L.

## 5 Results

### 5.1 Simulations under the statistical model

The variance of accuracy differences predicted by the model ($\sigma_{\bar{D}}^{2}$) was within 2% relative error of the Monte Carlo simulations across all simulation sets (RMS relative error 0.5%). The predicted power was within 4% error (simulated – predicted power) of the Monte Carlo simulations across all simulation sets with 95% confidence.

**Fig. 4:**
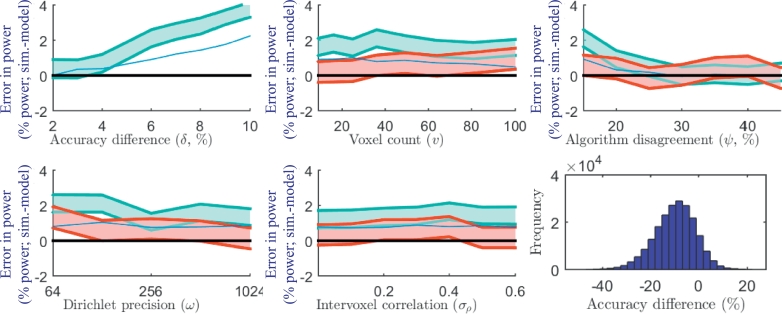
Model accuracy (95% confidence interval (shown in red for baseline δ=3% and in cyan for baseline δ=6%) on the absolute difference between the simulated and model power) for each simulation set. For example, with δ=10%, the model predicted 82% power, 4% below the 86% power observed in the simulation. Each accuracy graph shows a blue line representing the expected error due to the observed skew alone (for the simulation varying *δ* and the baseline δ=6%) based on applying the regular *t*-test sample size formula to a skewed Pearson distribution. The similar shape of this curve to the observed errors suggests that the skew is a considerable contributor to the error. The histogram (lower right) shows the distribution of accuracy differences for the simulation with δ=10%, illustrating the slight but significant skew in the distribution, which contributes to the observed error. (For interpretation of the references to colour in this figure legend, the reader is referred to the web version of this article.)

Fig. 4 shows the absolute error in the predicted power (i.e., simulation - model power) under varying model parameters. The parameter with the largest impact on the accuracy of power prediction was *δ*. For simulations with baseline δ=3% and δ=6%, the predicted power was within 2% and 3% absolute error, respectively, of the simulations with 95% confidence. A larger positive bias in the power prediction error across all values of *v, ω* and *σ_ρ_* was observed for simulations with δ=6%, compared to simulations with δ=3%, suggesting that the positive bias can be primarily attributed to the baseline accuracy difference. The simulation with δ=10% had the largest absolute error of 4%.

A proportion of the observed error can be attributed to skew in the distribution of per-image accuracy differences, deviating from the normality assumption of the *t*-test used in this work. The largest skew amongst our experiments (corresponding to the largest power prediction error) occurred when δ=10%; this is illustrated in a histogram of the accuracy differences, shown in Fig. 4. The effect of the deviation from normality is exacerbated in the simulations with large *δ* due to the lower sample size (n=8), for which the *t*-test is more sensitive to violations of its assumptions. To illustrate the expected impact of skew alone on the error in predicted power, Fig. 4 shows the error of the standard paired *t*-test power calculation for a correspondingly skewed population (Pearson distribution with skew matching the simulation) overlaid in blue.

**Fig. 5:**
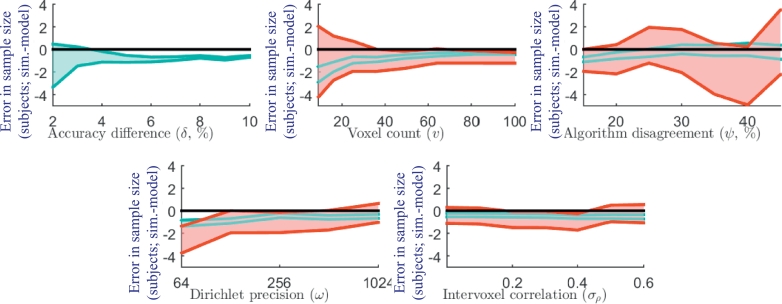
The equivalent error in predicted sample size (calculated from the observed error in power). Each plot shows the 95% confidence interval (shown in red for baseline δ=3% and in cyan for baseline δ=6%) on the absolute difference between the sample size needed to achieve the simulated power and the sample size needed to achieve the modeled power. For example, with δ=10%, the model would overestimate by 1 the number of subjects needed to achieve the 84% power observed in the simulation. (For interpretation of the references to colour in this figure legend, the reader is referred to the web version of this article.)

**Fig. 6:**
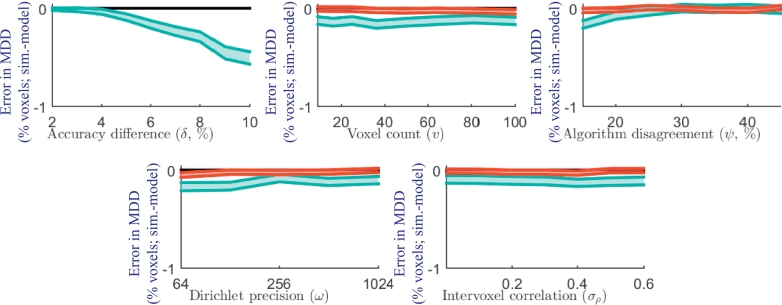
The equivalent error in predicted minimum detectable difference (calculated from the observed error in power). Each plot shows the 95% confidence interval (shown in red for baseline δ=3% and in cyan for baseline δ=6%) on the absolute difference between the minimum difference detectable with simulated power and the minimum difference detectable with the modeled power. For example, with δ=10%, the model would predict that a minimum detectable difference of 10.5% would result in the 84% power observed in the simulation. (For interpretation of the references to colour in this figure legend, the reader is referred to the web version of this article.)

The impact of these errors in predicted power on the sample size and minimum detectable difference is illustrated in Figs. 5 and 6.

### 5.2 Simulations with high-quality real-world data

When the minimum detectable difference was defined and tested relative to the high-quality reference standard in the PROMISE12 data set, the simulated power was  < 4% higher than the power specified by the model (approximately 80%) for the majority of algorithm comparisons (range 0–20%). The error was strongly correlated with the skew of per-image accuracy differences in the population (Spearman’s ρ=0.77; $p<1\times 10^{-8}$). The model did not over-estimate the power in any comparison, suggesting that it is conservative (i.e. avoiding predictions that result in underpowered studies) in the presence of skew. The errors for each pair of algorithms are reported in Table 5.

**Table 5:** Differences between the proportion of positive findings and the predicted power for simulated studies from the PROMISE12 data set using the high-quality reference standard. The required sample sizes predicted by the model are given in parentheses.

	B	C	D	E	F	G	H	I	J

A	3 (108)	1 (41)	12 (28)	2 (31)	14 (11)	1 (50)	2 (101)	13 (8)	7 (22)
B		10 (15)	1 (163)	1 (26418)	1 (35)	1 (1.8E6)	10 (28)	0 (14)	0 (157)
C			12 (11)	4 (10)	14 (9)	11 (12)	0 (42)	17 (5)	9 (6)
D				4 (102)	2 (50)	3 (115)	13 (14)	1 (15)	2 (3357)
E					7 (19)	1 (14084)	2 (11)	12 (8)	3 (95)
F						5 (23)	12 (10)	1 (312)	5 (48)
G							7 (16)	8 (10)	0 (97)
H								20 (5)	15 (8)
I									2 (17)

### 5.3 Simulations with low-quality real-world data

When the minimum detectable difference was defined relative to the high-quality reference standard and tested relative to the low-quality reference standard in the PROMISE12 data set, the model predicted the simulated power with a median error of 5% (simulated – predicted power; range -29–16%) and a median absolute error of 6% (|simulated – predicted power|). The two algorithm pairs with the smallest *δ_MDD_* (0.1% and 0.2% accuracy differences) and largest sample sizes (5714 and 3721) had the largest errors, overestimating power by 27% and 29%, respectively. The error was correlated with the skew of per-image accuracy differences (Spearman’s ρ=0.34; p=0.02), and excluding the 2 cases with the smallest *δ_MDD_*, the correlation was stronger (Spearman’s ρ=0.67; $p\approx 1\times 10^{-6}$). The errors for each pair of algorithms are reported in Table 6.

**Table 6:** Differences between the proportion of positive findings and the predicted power for simulated studies from the PROMISE12 data set using the low-quality reference standard. The required sample sizes predicted by the model are given in parentheses.

	B	C	D	E	F	G	H	I	J

A	6 (43)	−2 (167)	12 (22)	−5 (133)	2 (11)	−5 (25)	−27 (5714)	15 (7)	9 (21)
B		8 (14)	−6 (403)	8 (71)	−5 (67)	12 (3598)	12 (24)	−5 (17)	−29 (3721)
C			11 (12)	11 (34)	8 (11)	7 (13)	2 (50)	10 (6)	13 (8)
D				6 (31)	2 (87)	4 (165)	13 (16)	0 (17)	6 (508)
E					0 (15)	2 (41)	−1 (76)	11 (6)	6 (34)
F						0 (37)	5 (13)	4 (159)	4 (58)
G							4 (17)	6 (12)	−8 (466)
H								13 (6)	16 (11)
I									5 (16)

## 6 Case study

The direct application of the sample size formula to calculate the sample size is described in Section 3. The formula can also be used indirectly to guide other aspects in the design of segmentation comparison studies. In this case study, we illustrate one such application: evaluating the cost (in terms of sample size vs cost per subject) of using a lower-quality reference standard manually segmented by a non-clinical graduate student instead of one generated by clinical collaborators. For illustration, this case study simulates the availability of a pilot data set by using two algorithms and the 30 test data sets from the PROMISE12 challenge.

To evaluate the cost of the two approaches, we can compare the sample sizes under the two reference standard strategies. The error rates and minimum detectable difference *δ*_*MDD, H*_ will be the same for both scenarios. We use commonly accepted Type I and II error rates: α=0.05 and β=0.20. The appropriate *δ*_*MDD, H*_ depends on the clinical or technical requirements; for example, in the context of prostate segmentation, the MDD could represent the minimal improvement in prostate segmentation accuracy that would make an automated prostate MRI computer-aided detection (CAD) system (e.g. Litjens et al., 2014a) clinically suitable as a first reader. In this case study, we suppose that an analysis of an existing CAD system suggests an improvement in accuracy of 5% (with respect to a high-quality reference standard) would be sufficient to make the system clinically suitable.

The variance parameters differ between the scenarios. To assess the scenario where the study uses a high-quality reference standard, we can estimate $\hat{\psi },$$\hat{\delta }$ and ${\hat{\sigma }}_{\bar{D}}^{2}$ using A, B and H. Using Eqs. (11)–(13) with *h*_*k, i*_ in place of *l*_*k, i*_ gives $\hat{\psi }=13.4\%,$$\hat{\delta }=4.02\%$ and ${\hat{\sigma }}_{\bar{D}}^{2}=0.00231$. Since $\hat{\delta }$ and *δ_MDD_* are small relative to $\hat{\psi },$ assuming $\sigma_{0}^{2}=\sigma_{alt}^{2}={\hat{\sigma }}_{\bar{D}}^{2}$ will yield similar results to assuming a Dirichlet prior ($\sigma_{0}^{2}=0.00234$ and $\sigma_{alt}^{2}=0.00229$). The resulting sample size to detect a difference $\delta_{MDD,H}=5\%$ was 9 subjects. To assess the scenario where the study uses a low-quality reference standard instead, we first estimate ${\hat{\delta }}_{MDD}$ using A, B, L and H. Parameter estimation equations (Eqs. (9) and (10)) gives $\hat{p}\left(a\right)=0.246,$$\hat{p}\left(b\right)=0.195,$$\hat{p}\left(l\right)=0.210,$$\hat{p}\left(h\right)=0.214,$ and $\hat{cov}\left(A-B,L-H\right)=-0.29\%,$ yielding ${\hat{\delta }}_{MDD}=0.0348$. Using Eqs. (11)–(13) gives $\hat{\psi }=13.4\%,$$\hat{\delta }=3.37\%,$ and ${\hat{\sigma }}_{\bar{D}}^{2}=0.00253$. The resulting sample size to detect a difference $\delta_{MDD,H}=5\%$ was 12 subjects.

Based on this analysis, we estimate that a study using this lower-quality reference standard would require 30% more subjects to detect a 5% improvement in accuracy than one using the high-quality reference standard. Since the cost per subject of generating the lower-quality reference standard is typically much lower, this could be a suitable approach for comparing these algorithms.

## 7 Discussion

In this work, we derived a sample size formula for studies comparing the segmentation accuracy of two algorithms, and also a relationship describing the effect of using lower-quality reference standards on the minimum detectable difference in segmentation accuracy. The formula accuracy was evaluated using Monte Carlo simulations, yielding errors in predicted power of less than 4% across a range of model parameters. The applicability of the formulae to real-world data was evaluated using bootstrap sampling from the PROMISE12 prostate MRI segmentation data set yielding median errors in predicted power less than 6%, but showed the error to be sensitive to skewed distributions and small sample sizes. A case study was also analyzed to illustrate the use of the formulae in a realistic context.

### 7.1 Validation in segmentation comparison studies

Improvements in the methodology for the validation and comparison of segmentation algorithms span a wide variety of approaches.

One avenue to improve segmentation validation is to develop improved metrics. Simple segmentation metrics such as accuracy, Dice overlap, Cohen’s Kappa, mean absolute boundary distances and Hausdorff distances compare segmentations to a single reference standard and are commonly used (Taha and Hanbury, 2015). Newer metrics allow comparisons to multiple reference standards (e.g. the validation index (Juneja et al., 2013)) or comparisons that consider application specific utility (e.g. accuracy of quantitative measurements in segmented ROIs (Jha et al., 2012)). This latter concept can be taken further by validating segmentation through its impact on a larger system, such as the accuracy of a computer-assisted detection pipeline (Guo and Li, 2014). Model observers have also been developed to assess aspects of segmentation quality without a reference standard (Frounchi, Briand, Grady, Labiche, Subramanyan, 2011, Kohlberger, Singh, Alvino, Bahlmann, Grady, 2012); effectively creating a learned reference-standard-independent segmentation metric.

Another avenue to improve segmentation validation is to improve the reference standard quality. Label fusion algorithms, such as STAPLE (Warfield et al., 2004) and SIMPLE (Langerak et al., 2010) enable the generation of higher-quality reference standards that combine information from multiple experts. Improvements in multimodal registration (Shah, Pohida, Turkbey, Mani, Merino, Pinto, Choyke, Bernardo, 2009, Gibson, Crukley, Gaed, Gómez, Moussa, Chin, Bauman, Fenster, Ward, 2012) enable reference standards based on information that is less dependent on the image being segmented.

A third avenue is to increase the size of reference standards by reducing the cost per image, or via data augmentation. Active learning (Konyushkova, Sznitman, Fua, 2015, Top, Hamarneh, Abugharbieh, 2011) and other interactive annotation tools, reduce the cost of generating expert segmentations by partially automating the process. Crowdsourcing non-expert segmentations (Maier-Hein, Mersmann, Kondermann, Bodenstedt, Sanchez, Stock, Kenngott, Eisenmann, Speidel, 2014, Irshad, Montaser-Kouhsari, Waltz, Bucur, Nowak, Dong, Knoblauch, Beck, 2015) can cheaply generate many reference standards on many images, using the large numbers to offset the potential loss in quality. For some anatomy, artificial data with reference segmentations can be generated by simulating the imaging process (Cocosco et al., 1997) or perturbing the geometry and image signal of existing images (Hamarneh et al., 2008).

This work, in contrast, aims to improve validation by enabling researchers to design efficient and appropriately powered studies. This work focuses on a particular analysis used in segmentation comparison studies: comparing the proportion of voxels where each of two segmentation algorithms agree with a single reference standard. The presented formulae can be directly applied by researchers developing new segmentation algorithms to facilitate the design of their studies. More broadly, this work has particular importance for work focused on improving reference standard quality and reference standard size by providing a framework for understanding the tradeoffs between quality and quantity in segmentation reference standards.

### 7.2 Accuracy and applicability of the sample size formulae

In typical study designs, the statistical power, i.e. the probability of detecting an accuracy difference of a specified size, is fixed heuristically at 80%, specifying that a 20% risk of missing a true effect is acceptable. Other study design parameters are optimized under this constraint, balancing costs and effect sizes. A study design with statistical power substantially above the acceptable risk is using resource inefficiently, while one with lower power gives an unacceptable risk of false negatives. In our model, the largest errors observed in the model were for large accuracy differences. The variance predicted by the model matches the simulations to within 2%, suggesting that model errors are not primarily due to an incorrect variance prediction. Rather, the distribution of the accuracy differences in these simulation sets suggests that the error can be attributed to a combination of two factors: low sample size and skewness. The accuracy difference distribution under our statistical model, when using a Dirichlet prior, generally has non-zero skew when there are accuracy differences (i.e. |*δ*| > 0) and inter-image variability (*ω* < ∞), and the simulations show a skew as high as 0.3 in these simulation sets. The *t*-test, however, assumes samples are drawn from a normal distribution with 0 skew. While the *t*-test is robust to such deviations from normality at large sample sizes, large accuracy differences are more easily detectable and thus require small sample sizes. This suggests that segmentation comparison studies should be careful in their application of the *t*-test for studies with small sample sizes; in such cases, a McNemar test adjusted for clustered sampling (Gönen, 2004, Durkalski, Palesch, Lipsitz, Rust, 2003) may be more appropriate.

When applied to real-world data, the errors were generally larger than observed under the statistical model. The errors were strongly correlated with the skew of the distribution of per-image accuracy differences, which is consistent with our observations on simulated data. This effect was particularly evident when the predicted sample size was low: five of the six largest observed errors (where the model underestimated power by 13–20%) corresponded to simulated studies with *n* < 10, which is also consistent with our observations on simulated data. In general, the model underestimated the simulated power which could lead to inefficient resource usage, but would not lead to failed studies caused by insufficient power. When using a low-quality reference standard with *δ_MDD_* defined with respect to a high-quality reference standard, the error was also correlated with skew. However, in this context, another source of error must be considered: error in the estimation of *δ_MDD_*. When the estimated minimum detectable difference was very small ($|{\hat{\delta }}_{MDD}|<0.2\%$), small absolute estimation errors ($|\delta -{\hat{\delta }}_{MDD}|<0.06\%$) led to large relative estimation errors, resulting in large errors in the predicted power. When using a low-quality reference standard, the model over-estimated the simulated power for 10/45 of the algorithm pairs, suggesting that additional subjects may be needed when using this model to avoid underpowered studies.

The proposed approach for using low-quality reference standards presumes that a high-quality data set can be obtained, if only for a small pilot data set, and that clinical or technical requirements on accuracy differences specified with respect to that reference standard are useful. In some medical segmentation tasks (such as prostate cancer delineation on MRI (Gibson et al., 2016) or mitosis detection on histology images (Chowdhury et al., 2006)), even expert segmentations are highly variable. For some tasks, it may be appropriate to combine segmentations from multiple experts by consensus or using a label fusion algorithm such as STAPLE to generate a high-quality reference standard on a pilot study; however, care should be taken to consider whether requirements specified with respect to the resulting reference standard will be practically useful.

### 7.3 Model interpretation

Although the sample size relationship is a continuous function in multiple parameters, it can be useful to break the parameters into coarse categories to see emerging trends (see Table 7). In particular, we focus on the special case of modeling the prior as a Dirichlet random variable and examine the parameters that comprise the idealized efficiency *ψ*/*δ*^2^ and on the design factor *f*.

**Table 7:** Number of images required to detect a desired segmentation accuracy difference. When compensating for the use of a lower-quality reference standard, use Eq. (8) to estimate the minimum detectable difference (*δ_MDD_*) first.

		Design factor (*f*)		
		0.01	0.05	0.1
Small differences ($\delta_{MDD}=2\%$)				
ψ=2%	($\psi /\delta_{MDD}^{2}=50$)	6*	21	41
ψ=11%	($\psi /\delta_{MDD}^{2}=275$)	24	110	218
ψ=20%	($\psi /\delta_{MDD}^{2}=500$)	41	198	394
Medium differences ($\delta_{MDD}=5\%$)				
ψ=5%	($\psi /\delta_{MDD}^{2}=20$)	3*	10	17
ψ=12.5%	($\psi /\delta_{MDD}^{2}=50$)	6*	21	41
ψ=20%	($\psi /\delta_{MDD}^{2}=80$)	8*	33	65
Large differences ($\delta_{MDD}=10\%$)				
ψ=10%	($\psi /\delta_{MDD}^{2}=10$)	3*	6*	10
ψ=15%	($\psi /\delta_{MDD}^{2}=15$)	3*	8*	14
ψ=20%	($\psi /\delta_{MDD}^{2}=20$)	3*	10	17

* Small samples sizes calculated from Eq. (7) are reported here; however, studies with such small sample sizes may be highly sensitive to violations of the assumptions of the *t*-test, and are not recommended.

*δ_MDD_* can be coarsely categorized into small (*δ_MDD_* ≤ 2%), medium (2% < *δ_MDD_* < 10%), and large (*δ_MDD_* ≥ 10%) differences. Detecting small differences can require large (often infeasible) sample sizes, whereas detecting large differences may be limited not by *δ_MDD_* but by the assumptions of the statistical analysis.

Within these effect size categories, the likelihood of disagreement between algorithms (*ψ*) plays an important role. *ψ* has the range δ≤ψ≤δ+2min(p(A≠L),p(B≠L)). When *ψ* ≈ *δ*, it implies that most of the difference between the algorithm correspond to the more accurate algorithm correcting the errors of the less accurate one, while making few new errors. When *ψ* ≫ *δ*, the more accurate algorithm is making new errors on voxels where the less accurate algorithm was correct. Table 7 shows three levels of disagreement: minimal disagreement ($\psi =\delta_{MDD}$), large disagreement (ψ=20%) and a midpoint between them. When *δ_MDD_* is small, the level of disagreement can introduce an order of magnitude difference in required sample sizes.

The idealized efficiency is modulated by the design factor. The design factor ranges from 1/*v* (denoting that each voxel gives an independent estimate of accuracy differences) to 1 (denoting that each image gives an independent estimate of accuracy differences, but voxel segmentations are perfectly correlated). For realistic medical image segmentation algorithms, however, either of these extremes is unlikely. Table 7 shows three levels of the design factor: low correlation (f=0.01), medium correlation (f=0.05) and high correlation (f=0.1).

Our derivations show that sample sizes for studies comparing the accuracy of segmentation algorithms principally depend on the idealized efficiency $\psi /\delta_{MDD}^{2}$ which relates the probability of voxel-wise disagreement (*ψ*) between algorithms to the minimum detectable difference *δ_MDD_*, and the design factor *f* which reflects increased variability due to intervoxel correlation and inter-image variability. The sample size is approximately proportional to the idealized efficiency $\psi /\delta_{MDD}^{2}$. *ψ* has the range δ≤ψ≤δ+2min(p(A≠L),p(B≠L)), which suggests that it is easier, in general, to detect a given accuracy difference when at least one of the algorithms is highly accurate (lowering the upper bound on *ψ*). Furthermore, it is easier to detect a given accuracy improvement when algorithm A principally corrects errors made by algorithm B (where *ψ* ≈ *δ* minimizing the idealized efficiency) than when algorithm A has errors that are independent from B.

Although intuition would suggest that using lower-quality reference standards should consistently increase the required sample size, our derivations and simulations suggest a more complex relationship. The impact of errors in the reference standard is reduced by using a paired analysis which excludes variance due to factors that affect both algorithms in the same way, such as reference standard errors in voxels where the algorithms agree. Reference standard errors in regions of disagreement, however, do affect the variance of per-image accuracy differences ($\sigma_{\bar{D}}^{2}=\frac{1+\omega \bar{\rho_{i,j}}}{\omega +1}\left(\psi -\delta^{2}\right)$ from Eq. (6)). In the rightmost term of this equation, *ψ* (which does not depend on the reference standard) is generally much larger than *δ*^2^ (see Table 7), suggesting that the impact of reference standard errors on variance is predominantly via changing the design factor. Reference standard errors also affect the sample size (Eq. (8)) by altering the detectable accuracy difference when the reference standard has errors that are biased in favour of one algorithm or when it has systematic over- or under contouring *and* one algorithm contours more foreground than the other. Relatively speaking, systematic over- or under contouring will have only a small impact on the detectable accuracy differences, unless the algorithms’ foreground proportions are very different: for example, if A contours 5% more foreground than B, then 10% over-contouring by L (25 ×  that observed in the PROMISE12 data) will change the measured accuracy difference by only 0.5%, unless the contouring errors are biased towards one algorithm. Furthermore, errors in the reference standard that are biased towards one algorithm do not necessarily decrease power: reference standard errors biased towards the more accurate algorithm will exaggerate the true difference, increasing power at the expense of increased type I error.^1^ These observations were reflected in our analysis of the PROMISE12 challenge data (see Tables 5 and 6). Comparing the low-quality to the high-quality reference standard, the root-mean-squared relative error in $\hat{f}$ was 4%, compared to 0.3% for $\hat{\psi }-{\hat{\delta }}^{2}$. Because the low-quality reference standard had substantial agreement with the high-quality one (96% ± 1% mean ± SD accuracy), the effect of sample biases in reference standard errors were observable: for 17/45 pairs of algorithms, the studies designed to use the low-quality reference standard actually needed fewer subjects than studies using the high-quality reference standard; in all of these cases, there were slight sample biases in the low-quality reference standard towards the more accurate algorithm (primarily, as expected, in the covariance term in Eq. (8)). This increased |*δ_MDD_*| relative to |*δ_H_*| (i.e. the underlying differences between the algorithms were exaggerated and thus easier to detect). Because the experimental design for evaluating the model on real data required $\delta_{MDD}=\delta_{H},$ which was very small for some comparisons ( < 2% in 20/45 algorithm pairs and  < 0.5% in 4 algorithm pairs), this effect was magnified. Overall, our analysis of the PROMISE12 data aligns well with our theoretical model. Based on our analysis, using reference standards that are lower quality but unbiased may be a suitable approach for comparing segmentation algorithm accuracy.

### 7.4 Limitations

The contributions of this work should be considered in the context of its limitations. First, the sample size calculation presented in this work is specific to the statistical analysis (the paired Student’s *t*-test) and to the accuracy metric (proportion of voxels matching the reference standard). Further work is needed to develop these formulae for other analyses and metrics. Second, our correlation model is over-parameterized, representing inter-image variability and intra-image inter-voxel correlation separately, when their effect on the covariance of ${\vec{D}}_{k}$ is coupled. This complicates the estimation of parameters, but yields formulae expressed in concepts familiar to the image analysis community. Third, due to constraints on sampling from specified high-dimensional correlated discrete distributions, we were unable to generate Monte Carlo simulations testing the extremes of some parameter ranges (e.g. high numbers of voxels and high intervoxel correlation). Because the metric analysed in the study ${\bar{D}}_{k}$ is a mean over voxels (which becomes more precise with higher *v*) and because we did not observe an increase in error as *v* increased from 9–100, we do not anticipate notable differences in model performance with larger *v*. Fourth, our application of the formulae to real segmentation studies was limited by the public availability of data sets with high- and low- quality reference standards; the PROMISE12 data set used in our case study is a rare example of such data. Finally, the sensitivity of the formula to violations of its underlying assumptions was not estimated; future work in this area could clarify which of these assumptions are critical to the accuracy of the formula and which could be relaxed.

## 8 Conclusions

In this work, we derived formulae to address two interrelated questions in the design of studies comparing segmentation algorithms: How many validation images are needed to evaluate a segmentation algorithm? and How accurate does the reference standard need to be? The sample size formula predicted the power of simulated segmentation studies to within 4% across a range of model parameters, and when applied to the PROMISE12 prostate segmentation challenge data, predicted the power to within a median error of 6%. In addition to their direct application in calculating sample sizes, the formulae offer several insights for study design. First, it is generally easier to detect a given accuracy difference when at least one algorithm is highly accurate, as this reduces accuracy variability. Second, it is generally easier to detect a given accuracy difference when one algorithm principally corrects the errors of another, compared to when two algorithm make independent errors. Third, systematic over- or under-contouring by a low-quality reference standard does not impact accuracy measurements substantially unless one algorithm tends to contour more voxels as foreground than the other, but correlation between reference standard errors and algorithm differences can bias accuracy measurements. These formulae, and parameter estimation equations and guidelines that facilitate their use, hold the potential to enable researchers to make statistically motivated decisions about their study design and their choice of reference standard and to make the most efficient use of limited research resources.

## Appendix A Derivation of the variance of the accuracy difference

The variance of the per-image difference in accuracy $\sigma_{\bar{D}}^{2}$ affects the statistical power of segmentation accuracy comparison experiments. This appendix derives an expression for $\sigma_{\bar{D}}^{2}$ based on the statistical model described in Section 2 for any prior distribution of per-image average marginal probabilities (${\vec{O}}_{k}∼P\left(\vec{p}\right)$) in terms of moments of the prior distribution.

### A1 Statistical segmentation model and notation reiterated

The per-image difference in accuracy is ${\bar{D}}_{k}=\frac{1}{v}\sum_{i=1}^{v}D_{k,i},$ where *v* is the number of voxels and random variable *D*_*k, i*_ is the per-voxel segmentation accuracy difference for the *i*th voxel in the *k*th image defined as $D_{k,i}=|B_{k,i}-L_{k,i}\left|-\right|A_{k,i}-L_{k,i}|$. Random variables *A*_*k, i*_, *B*_*k, i*_ and *L*_*k, i*_ are segmentation labels for the *i*th voxel in the *k*th image from segmentation algorithms A and B and reference standard L, respectively.

The statistical model, motivated and described in Section 2, models the distribution of the random vector of per-voxel accuracy differences ${\vec{D}}_{k}=\langle D_{k,1},\ldots ,D_{k,v}\rangle$ as a *v*-dimensional correlated categorical distribution with three categories (1, 0, and −1). The marginal probabilities ${\vec{O}}_{k,i}=<O_{k,i,1},O_{k,i,0},O_{k,i,-1}>$ of the categorical distribution are identically distributed random probability vectors with mean ${\vec{O}}_{k}=<O_{k,1},O_{k,0},O_{k,-1}>,$ but no other constraint on the shape of the distribution. The covariance of the categorical distribution given ${\vec{O}}_{k}$ is defined such that $cov\left(D_{k,i},D_{k,j}|{\vec{O}}_{k}\right)=\rho_{i,j}\sqrt{\sigma_{D_{k,i}|{\vec{O}}_{k}}^{2}\sigma_{D_{k,j}|{\vec{O}}_{k}}^{2}},$ where $\sigma_{D_{k,i}|{\vec{O}}_{k}}^{2}$ is the conditional variance of *D*_*k, i*_ given ${\vec{O}}_{k}$. Priors ${\vec{O}}_{k}$ are independently and identically distributed random variables sampled for each image with mean $\vec{p}$ (the population mean probability vector).

### A2 Derivation of $\sigma_{\bar{D}}^{2}$ in terms of moments of priors ${\vec{O}}_{k}$

To derive $\sigma_{\bar{D}}^{2}$ under this model, we express the covariance matrix of variables *D*_*k, i*_ in terms of $E\left(D_{k,i}|{\vec{O}}_{k,i}\right)=O_{k,i,1}-O_{k,i,-1}$ and $E\left(D_{k,i}^{2}|{\vec{O}}_{k,i}\right)=O_{k,i,1}+O_{k,i,-1},$ marginalize out prior parameters ${\vec{O}}_{k}$ and $\vec{O_{k,i}}$ to give an expression in terms of moments of ${\vec{O}}_{k},$ and express $\sigma_{\bar{D}}^{2}$ as the average of covariance matrix elements.

Because ${\bar{D}}_{k}=\frac{1}{v}\sum_{i=1}^{v}D_{k,i},$$\sigma_{\bar{D}}^{2}$ can be expressed as

(A.1) $$\begin{array}{c} \sigma_{\bar{D}}^{2}=\frac{1}{v^{2}}\sum_{i,j}cov\left(D_{k,i},D_{k,j}\right), \end{array}$$

for a random image *k*. By the law of total covariance, *cov*(*D*_*k, i*_, *D*_*k, j*_) can be expressed in terms of conditional probabilities given ${\vec{O}}_{k}$ as the sum of two components,

(A.2) $$\begin{array}{ccc} \sigma_{\bar{D}}^{2} & = & \frac{1}{v^{2}}\sum_{i,j}cov\left(E\left(D_{k,i}|{\vec{O}}_{k}\right),E\left(D_{k,j}|{\vec{O}}_{k}\right)\right)\\ & & +\;E[cov\left(D_{k,i},D_{k,j}|{\vec{O}}_{k}\right)], \end{array}$$

where *E*(*X*|*Y*) denotes the conditional expectation of *X* given *Y*, and *cov*(*X, Y*|*Z*) denotes the conditional covariance of *X* and *Y* given *Z*. The two components can be expressed in terms of moments of ${\vec{O}}_{k}$ by

1. expressing each component in terms of marginal probabilities ${\vec{O}}_{k,i},$
2. marginalizing them over ${\vec{O}}_{k,i}$ to express them in terms of ${\vec{O}}_{k}$ and
3. marginalizing them over ${\vec{O}}_{k}$ to express them in terms of moments of ${\vec{O}}_{k}$.

It is helpful to first note that $E\left(D_{k,j}|{\vec{O}}_{k}\right)=E\left(D_{k,i}|{\vec{O}}_{k}\right)$ and $\sigma_{D_{k,i}|{\vec{O}}_{k}}^{2}=\sigma_{D_{k,j}|{\vec{O}}_{k}}^{2},$ since ${\vec{O}}_{k,i}$ and ${\vec{O}}_{k,j}$ are identically distributed given ${\vec{O}}_{k}$. The first term of Eq. (A.2) represents the covariance due to variability of the prior, and can be simplified following the three steps above (shown in Eqs. (A.3), (A.4) and (A.5)) with details shown below:

(A.3) $$\begin{array}{ccc} & & cov(E\left(D_{k,i}|{\vec{O}}_{k}\right),E\left(D_{k,j}|{\vec{O}}_{k}\right))\\ & & \;=var(E\left(D_{k,i}|{\vec{O}}_{k}\right))\\ & & \;=var\left(\int E\left(D_{k,i}|{\vec{O}}_{k,i}\right)p\left({\vec{O}}_{k,i}|{\vec{O}}_{k}\right)d{\vec{O}}_{k,i}\right) \end{array}$$

(A.4) $$\begin{array}{ccc} & & \;=var\left(\int \left(O_{k,i,1}-O_{k,i,-1}\right)p\left({\vec{O}}_{k,i}|{\vec{O}}_{k}\right)d{\vec{O}}_{k,i}\right)\\ & & \;=var(O_{k,1}-O_{k,-1}) \end{array}$$

(A.5) $$\begin{array}{c} \;=\sigma_{O_{1}}^{2}+\sigma_{O_{-1}}^{2}-2\sigma_{O_{1},O_{-1}}, \end{array}$$

where $\sigma_{O_{1}}^{2}$ and $\sigma_{O_{-1}}^{2}$ are the variances of *O*_1_ and $O_{-1}$ and $\sigma_{O_{1},O_{-1}}$ is the covariance of *O*_*k*, 1_ and $O_{k,-1}$.

The second component of Eq. (A.2) represents the covariance due to sampling the marginal probability and per-voxel accuracy difference variables, and can be simplified following the three steps above (shown in Eqs. (A.6), (A.7) and (A.8)) with details shown below:

(A.6) $$\begin{array}{ccc} & & E[cov\left(D_{k,i},D_{k,j}|{\vec{O}}_{k}\right)]\\ & & \;=E[\rho_{i,j}\sigma_{D_{k,i}|{\vec{O}}_{k}}\sigma_{D_{k,j}|{\vec{O}}_{k}}]\\ & & \;=E[\rho_{i,j}\sigma_{D_{k,i}|{\vec{O}}_{k}}^{2}]\\ & & \;=\rho_{i,j}E\left[E\left(D_{k,i}^{2}|{\vec{O}}_{k}\right)-E{\left(D_{k,i}|{\vec{O}}_{k}\right)}^{2}\right]\\ & & \;=\rho_{i,j}E[\int E\left(D_{k,i}^{2}|{\vec{O}}_{k,i}\right)p\left({\vec{O}}_{k,i}|{\vec{O}}_{k}\right)d{\vec{O}}_{k,i}\\ & & \;-{\left(\int E\left(D_{k,i}|{\vec{O}}_{k,i}\right)p\left({\vec{O}}_{k,i}|{\vec{O}}_{k}\right)d{\vec{O}}_{k,i}\right)}^{2}] \end{array}$$

(A.7) $$\begin{array}{ccc} & & \;=\rho_{i,j}E[\int \left(O_{k,i,1}+O_{k,i,-1}\right)p\left({\vec{O}}_{k,i}|{\vec{O}}_{k}\right)d{\vec{O}}_{k,i}\\ & & \;-{\left(\int \left(O_{k,i,1}-O_{k,i,-1}\right)p\left({\vec{O}}_{k,i}|{\vec{O}}_{k}\right)d{\vec{O}}_{k,i}\right)}^{2}]\\ & & \;=\rho_{i,j}E\left[O_{k,1}+O_{k,-1}-{\left(O_{k,1}-O_{k,-1}\right)}^{2}\right] \end{array}$$

(A.8) $$\begin{array}{ccc} & & \;=\rho_{i,j}(E\left[O_{k,1}\right]+E\left[O_{k,-1}\right]-E\left[O_{k,1}^{2}\right]\\ & & \;+\;2E\left[O_{k,1}O_{k,-1}\right]-E\left[O_{k,-1}^{2}\right])\\ & & \;=\rho_{i,j}\left(p_{1}+p_{-1}-E\left[O_{k,1}^{2}\right]+2E\left[O_{k,1}O_{k,-1}\right]-E\left[O_{k,-1}^{2}\right]\right)\\ & & \;=\rho_{i,j}(p_{1}+p_{-1}-\left(\sigma_{O_{1}}^{2}+\mu_{O_{1}}^{2}\right)\\ & & \;+\;2\left(\sigma_{O_{1},O_{-1}}+\mu_{O_{1}}\mu_{O_{-1}}\right)-\left(\sigma_{O_{-1}}^{2}+\mu_{O_{-1}}^{2}\right))\\ & & \;=\rho_{i,j}\left(p_{1}+p_{-1}-\sigma_{O_{1}}^{2}+2\sigma_{O_{1},O_{-1}}-\sigma_{O_{-1}}^{2}-{\left(p_{1}-p_{-1}\right)}^{2}\right). \end{array}$$

Substituting Eqs. (A.5) and (A.8) into Eq. (A.2) yields the variance of the per-image accuracy in terms of moments of the prior ${\vec{O}}_{k}$:

(A.9) $$\begin{array}{ccc} \sigma_{\bar{D}}^{2} & = & \bar{\rho_{i,j}}\left(p_{1}+p_{-1}-{\left(p_{1}-p_{-1}\right)}^{2}\right)\\ & & +\;\left(1-\bar{\rho_{i,j}}\right)\left(\sigma_{O_{1}}^{2}-2\sigma_{O_{1},O_{-1}}+\sigma_{O_{-1}}^{2}\right), \end{array}$$

where $\bar{\rho_{i,j}}=\frac{1}{v^{2}}\sum_{i,j}\rho_{i,j}$ is the average of the intra-image inter-voxel correlation coefficients. For conciseness, we introduce two terms: $\psi =p_{1}+p_{-1}$ is the population-wide probability that algorithms A and B disagree on the labeling of a voxel, and $\sigma_{O_{1}-O_{-1}}^{2}=\sigma_{O_{1}}^{2}-2\sigma_{O_{1},O_{-1}}+\sigma_{O_{-1}}^{2}$ is the variance of $O_{1}-O_{-1}$ for the prior ${\vec{O}}_{k}$. This substitution yields a more concise expression (identical to Eq. (4)):

(A.10) $$\begin{array}{c} \sigma_{\bar{D}}^{2}=\bar{\rho_{i,j}}\left(\psi -\delta^{2}\right)+\left(1-\bar{\rho_{i,j}}\right)\sigma_{O_{1}-O_{-1}}^{2}. \end{array}$$

## Appendix B Derivation of the accuracy difference in terms of the high-quality reference standard

The minimum detectable difference *δ_MDD_* must be defined with respect to the study’s reference standard, while clinical or technical requirements may be better defined with respect to a high-quality reference standard (*δ*_*MDD, H*_). This appendix derives an equation to express the population average accuracy difference with respect to one reference standard (L) as a function of the population average accuracy difference with respect to another reference standard (H), and uses this to express *δ_MDD_* as a function of *δ*_*MDD, H*_ when a low-quality reference standard is used.

### B1 Model and notation

As we did for A, B and L, we consider the segmentation labels of the high-quality reference standard H as random variables. We denote the population average accuracy difference with respect to L as *δ*, and that with respect to H as *δ_H_*. We abbreviate the probability of a particular combination of segmentation labels for a randomly selected voxel as the conjunction of events $\bar{a},$$\bar{b},$$\bar{l}$ and $\bar{h}$ when the respective labels are 0 and **a, b, l** and **h** when the respective labels are 1. For example, $p(a\bar{b}l)$ denotes the probability that A gives the label 1, B gives the label 0 and L gives the label 1 for the randomly selected voxel.

### B2 Derivation

As described in Section 2.4, the derivation of *δ* as a function of *δ_H_* uses the following approach:

1. Express *δ* in terms of the joint probability of segmentation labels of A, B, L and H
2. Isolate the terms of this expression that equate to *δ_H_*, and simplify the remaining terms

### B3 Express *δ* in terms of the joint probability of segmentation labels of A, B, L and H

Since events where A=B do not affect the *difference* in accuracy, *δ* is the probability of events where A=L and *B* ≠ *L* minus the probability of events where *A* ≠ *L* and B=L. *δ* can be expressed in terms of the probabilities of specific combinations of segmentation labels for A, B and L for a randomly selected voxel:

(B.1) $$\begin{array}{c} \delta =p\left(a\bar{b}l\right)+p\left(\bar{a}b\bar{l}\right)-p\left(\bar{a}\mathrm{bl}\right)-p\left(a\bar{b}\bar{l}\right). \end{array}$$

We then express each term in Eq. (B.1) in terms of H with a substitution $p\left(\mathrm{xy}\right)=p\left(\mathrm{xz}\right)-p\left(x\bar{y}z\right)+p\left(\mathrm{xy}\bar{z}\right),$ where **x** represents $a\bar{b}$ (for term 1 and 4) or $\bar{a}b$ (for term 2 and 3) and **y** and **z** represent **l** and **h** (for term 1 and 3) or $\bar{l}$ and $\bar{h}$ (for term 2 and 4):

(B.2) $$\begin{array}{ccc} \delta & = & \left(p\left(a\bar{b}h\right)-p\left(a\bar{b}\bar{l}h\right)+p\left(a\bar{b}l\bar{h}\right)\right)\\ & & +\;(p\left(\bar{a}b\bar{h}\right)-p\left(\bar{a}\mathrm{bl}\bar{h}\right)+p\left(\bar{a}b\bar{l}h\right))\\ & & -\;(p\left(\bar{a}\mathrm{bh}\right)-p\left(\bar{a}b\bar{l}h\right)+p\left(\bar{a}\mathrm{bl}\bar{h}\right))\\ & & -\;(p\left(a\bar{b}\bar{h}\right)-p\left(a\bar{b}l\bar{h}\right)+p\left(a\bar{b}\bar{l}h\right)). \end{array}$$

### B4 Isolate the terms of this expression that equate to *δ_H_*, and simplify the remaining terms

The difference in accuracy with respect to H is $\delta_{H}=p\left(a\bar{b}h\right)+p\left(\bar{a}b\bar{h}\right)-p\left(\bar{a}\mathrm{bh}\right)-p\left(a\bar{b}\bar{h}\right)$. Isolating these terms in Eq. (B.2) gives the sum of *δ_H_* and an error term:

(B.3) $$\begin{array}{c} \delta =\delta_{H}+2\left(p\left(a\bar{b}l\bar{h}\right)-p\left(a\bar{b}\bar{l}h\right)+p\left(\bar{a}b\bar{l}h\right)-p\left(\bar{a}\mathrm{bl}\bar{h}\right)\right). \end{array}$$

To simplify the error term, we first expand each term with the substitution $p\left(x\bar{y}\bar{z}\right)=p\left(x\right)-p\left(\mathrm{xyz}\right)-p\left(\mathrm{xy}\bar{z}\right)-p\left(x\bar{y}z\right),$ where **x** represents the non-complemented terms, and $\bar{y}$ and $\bar{z}$ represent the complemented terms, giving

(B.4) $$\begin{array}{ccc} \delta & = & \delta_{H}+2\left(p\left(\mathrm{al}\right)-p\left(\mathrm{ablh}\right)-p\left(a\bar{b}\mathrm{lh}\right)-p\left(\mathrm{abl}\bar{h}\right)\right)\\ & & -\;2(p\left(\mathrm{ah}\right)-p\left(\mathrm{ablh}\right)-p\left(a\bar{b}\mathrm{lh}\right)-p\left(\mathrm{ab}\bar{l}h\right))\\ & & +\;2(p\left(\mathrm{bh}\right)-p\left(\mathrm{ablh}\right)-p\left(\bar{a}\mathrm{blh}\right)-p\left(\mathrm{ab}\bar{l}h\right))\\ & & -\;2(p\left(\mathrm{bl}\right)-p\left(\mathrm{ablh}\right)-p\left(\mathrm{abl}\bar{h}\right)-p\left(\bar{a}\mathrm{blh}\right)), \end{array}$$

and then cancel out duplicated terms, giving

(B.5) $$\begin{array}{c} \delta =\delta_{H}+2\left(p\left(\mathrm{al}\right)-p\left(\mathrm{ah}\right)+p\left(\mathrm{bh}\right)-p\left(\mathrm{bl}\right)\right). \end{array}$$

If *A, B, L*, and *H* are encoded as 0 (for background) and 1 (for foreground), this error term can be expressed as 2(p(a)−p(b))(p(l)−p(h))+2cov(A−B,L−H) in the full equation:

(B.6) $$\begin{array}{c} \delta =\delta_{H}+2\left(p\left(a\right)-p\left(b\right)\right)\left(p\left(l\right)-p\left(h\right)\right)+2cov\left(A\;-\;B,L\;-\;H\right). \end{array}$$

By substituting $\delta =\delta_{MDD}$ and $\delta_{H}=\delta_{MDD,H}$ into Eq. (B.6), we can express *δ_MDD_* as a function of *δ*_*MDD, H*_ when a low-quality reference standard is used (identical to Eq. (8)):

(B.7) $$\begin{array}{ccc} \delta_{MDD} & = & \delta_{MDD,H}+2\left(p\left(a\right)-p\left(b\right)\right)\left(p\left(l\right)-p\left(h\right)\right)\\ & & +\;2cov(A-B,L-H). \end{array}$$

## Appendix C Supplementary materials

Supplementary Data S1 Supplementary Raw Research Data. This is open data under the CC BY license http://creativecommons.org/licenses/by/4.0/ Supplementary Data S1

## Footnotes

1 Because of this, care should be taken when estimates of this bias (Eq. (10)) are not substantially smaller than *δ_H_*.
