# Supplementary material for: Designing image segmentation studies: Statistical power, sample size and reference standard quality
Source: Med Image Anal. 2017 Dec;42:44–59. doi: 10.1016/j.media.2017.07.004 (PMC5666910; doi:10.1016/j.media.2017.07.004)
Supplement: Supplementary Data S1 — Supplementary Raw Research Data. This is open data under the CC BY license http://creativecommons.org/licenses/by/4.0/ [file mmc1.pdf]

Designing image segmentation studies: statistical power, sample size and reference standard quality: Supplementary material

Eli Gibson[a,b,*], Yipeng Hu[b], Henkjan J. Huisman[a], Dean C. Barratt[b]

[a] Department of Radiology, Radboud University Medical Center, Nijmegen, The Netherlands
[b] Department of Medical Physics and Biomedical Engineering, University College London, London, United Kingdom

Abstract

This supplementary material contains the formulae for the sensitivity of the Dirichlet-based sample size formula to parameter estimation error.

1. Sensitivity of predicted sample sizes to parameter estimation error

Parameters in sample size calculations are typically estimated from literature or from pilot data. Errors in these parameters can propagate to errors in the predicted sample size requirements. Understanding the sensitivity of sample size predictions to parameter estimation errors can inform decisions about sample size safety margins in designing studies, particularly when combined with estimates of the precision of the parameter estimators.

Under the assumption of a Dirichlet-distributed prior on the average marginal probabilities, we can find a linear approximation of the sensitivity by taking the derivative of the Dirichlet-prior-based sample size formula (identical to Equation 7),

$$n = \frac{1+\omega\overline{\rho_{i,j}}}{\omega+1}\left(t_{\alpha/2}\sqrt{\psi/\delta_{MDD}^2}+t_\beta\sqrt{\psi/\delta_{MDD}^2-1}\right)^2. \quad (1)$$

with respect to the estimated parameters δ_{MDD}, ψ, $\overline{\rho_{i,j}}$, and ω. To simplify the expressions, we express each equation in terms of relative errors and also make a substitution $k = \frac{\sqrt{\psi/\delta_{MDD}^2-1}}{\sqrt{\psi/\delta_{MDD}^2}}$.

$$\frac{dN/N}{d\overline{\rho_{i,j}}/\overline{\rho_{i,j}}} = \frac{\overline{\rho_{i,j}}\omega}{\overline{\rho_{i,j}}\omega+1} \quad (2)$$

$$\frac{dN/N}{d\omega/\omega} = -\frac{\omega(1-\overline{\rho_{i,j}})}{(\overline{\rho_{i,j}}\omega+1)(\omega+1)} \quad (3)$$

$$\frac{dN/N}{d\psi/\psi} = \frac{T_{\alpha\{2\}}+T_{\beta\{1\}}/k}{T_{\alpha\{2\}}+T_{\beta\{1\}}k} \quad (4)$$

$$\frac{dN/N}{d\delta_{MDD}/\delta_{MDD}} = -2\frac{T_{\alpha\{2\}}+T_{\beta\{1\}}/k}{T_{\alpha\{2\}}+T_{\beta\{1\}}k}. \quad (5)$$

Substituting estimated parameter values into these equations, and multiplying the result by the relative error in the parameter being evaluated gives an estimate of the expected relative error in the sample size.

*Corresponding author
Email: eli.gibson@ucl.ac.uk
Mailing Address:
Centre for Medical Image Computing
The Engineering Front Building
University College London
Malet Place
London, WC1E 6BT
